# Beetroot leaf extract enhances gut motility in a model of loperamide‐induced constipation in male rats: A phytopharmacological study

**DOI:** 10.14814/phy2.70467

**Published:** 2025-09-09

**Authors:** Ala Ayari, Saber Jedidi, Houcem Sammari, Nourhene Dhawefi, Soumaya wahabi, Hichem Sebai

**Affiliations:** ^1^ University of Jendouba, Laboratory of Functional Physiology and Valorization of Bio‐Resources, Higher Institute of Biotechnology of Béja Béja Tunisia; ^2^ University of Jendouba, National Institute of Technologies and Sciences of Kef (NITeK) Le Kef Tunisia

**Keywords:** beetroot leaves, constipation, gastrointestinal motility, inflammation, oxidative stress, rats

## Abstract

Constipation is a common gastrointestinal disorder characterized by infrequent and difficult bowel movements, hard stool consistency, and delayed intestinal transit. The present study evaluated the phytochemical profile and physiological effects of the aqueous extract of beetroot leaves (AEBL) in a rat model of Loperamide (LOP)‐induced constipation. Thirty‐six male *Wistar* rats were randomly assigned to six groups (*n* = 6): two controls (normal and constipated) and four constipated groups receiving either increasing doses of AEBL (100, 200, or 400 mg/kg, *b.w*.) or yohimbine (YOH; 2 mg/kg, *b.w*.) as a reference prokinetic agent. Treatments were administered orally 1 h after LOP intoxication for seven consecutive days. The phytochemical screening revealed that AEBL is rich in polyphenols, flavonoids, and betalains. Antioxidant assays (DPPH and ABTS) demonstrated strong radical scavenging activity. In vivo, the oral administration of AEBL showed no signs of toxicity or abnormal behavior in rats, with an LD_50_ greater than 2000 mg/kg *bw*. Additionally, AEBL significantly improved stool frequency and gastrointestinal motility, reduced oxidative stress, restored antioxidant defenses, rebalanced serum electrolytes, and attenuated LOP‐induced inflammation. Histological analysis confirmed enhanced mucosal integrity and preservation of colonic architecture. These results support the potential of beetroot leaf extract as a natural, multi‐targeted therapeutic strategy for alleviating constipation and associated gastrointestinal dysfunctions.

## 1 INTRODUCTION

The use of medicinal plants for therapeutic purposes, a practice known as phytotherapy, has been an integral part of traditional medicine across various cultures for centuries (de Carvalho Nilo Bitu et al., 2015). Among these medicinal plants, beetroot (*Beta vulgaris* L.) stands out as a widely cultivated and consumed vegetable, particularly in the Mediterranean region. Belonging to the *Amaranthaceae* family, beetroot is primarily cultivated for its edible root, which is known for its rich nutrient profile and health benefits (Punia Bangar et al., 2022; Thiruvengadam et al., 2024).

Beetroot is recognized for its exceptional nutritional properties, making it a valuable addition to a healthy diet (Moulick et al., 2023; Sadowska‐Bartosz & Bartosz, 2021). The roots are rich in essential vitamins, including vitamin A, vitamin C, vitamin B6, and folate. In addition, this plant is a source of key minerals such as iron, manganese, potassium, and magnesium. These vitamins and mineral matter play a critical role in maintaining various bodily functions, including immune support, cellular health, and skin integrity (Rehman et al., 2021) and beneficial effects for cardiovascular health, bone development, and cellular function (Chhikara et al., 2019). Furthermore, beetroot is particularly noted for its high fiber content and its antioxidant compounds, such as betacyanins, which are responsible for the plant's characteristic vivid red color. These compounds not only provide color but also contribute to its beneficial effects on health, particularly in the management of various gastrointestinal disorders (Calvi et al., 2023; Rahimi et al., 2019).

However, the leaves of beetroot, often overlooked, also offer significant nutritional and therapeutic value (Rehman et al., 2021). Notably, beetroot leaves are rich in bioactive compounds including polyphenols, flavonoids, and dietary fibers, which are associated with antioxidant, anti‐inflammatory, and cytoprotective effects (De Oliveira et al., 2021; Moulick et al., 2023; Rehman et al., 2021). Despite their phytochemical richness, studies evaluating the effects of beetroot leaves on gastrointestinal health remain scarce. De Oliveira et al. (2021) highlighted the potential of *Beta vulgaris* leaves to modulate gut microbiota, yet experimental in vivo studies specifically focusing on beetroot leaf extract in constipation models are lacking. This gap underscores the need for research to explore the protective and pro‐motility effects of beetroot leaf extract in digestive disorders.

Gastrointestinal disorders encompass a wide range of conditions that affect the digestive tract, disrupting normal digestion, and absorption of nutrients (Greenwood‐Van Meerveld et al., 2017). Among these disorders, constipation is one of the most prevalent and debilitating conditions. Characterized by infrequent, difficult, or painful bowel movements, constipation is a common problem that affects a large proportion of the global population. Although the frequency of bowel movements can vary from person to person, constipation is generally defined as having fewer than three bowel movements per week, accompanied by discomfort, abdominal bloating, and a feeling of incomplete evacuation (Ayari, Dakhli, et al., 2025; Ayari, Jedidi, et al., 2025; Liu et al., 2025).

Several factors contribute to the development of constipation, which can range from mild to chronic. Poor dietary habits, such as low fiber intake, insufficient fluid consumption, and sedentary lifestyles, are often linked to the onset of constipation (Wang et al., 2024). Additionally, certain medications, such as opioids, antacids, and iron supplements, are well known to disrupt normal bowel function (Aziz et al., 2020). Chronic constipation can cause significant discomfort and lead to complications such as hemorrhoids, anal fissures, and fecal impaction, all of which can severely affect a person's quality of life (Ayari, Dakhli, et al., 2025; Ayari, Jedidi, et al., 2025).

In light of these challenges, there has been a growing interest in seeking natural alternatives to treat constipation (Akram et al., 2022; Huang et al., 2024). Conventional laxatives, while effective in the short term, are often associated with side effects such as abdominal cramps, dehydration, and the risk of dependency with prolonged use (Pergolizzi Jr et al., 2017). Consequently, there is increasing interest in safer, sustainable alternatives such as medicinal plants (Paknejad et al., 2019).

This study therefore aims to investigate, for the first time, the nutritional value, antioxidant potential, and in vivo protective effects of beetroot leaves aqueous extract in a rat model of loperamide‐induced constipation, focusing on improvements in gastrointestinal motility, reduction of oxidative stress, and restoration of inflammatory biomarkers.

## 2 MATERIALS AND METHODS

### 2.1 Reagents and chemicals

Gallic acid (PubChem CID: 370), ascorbic acid (PubChem CID: 54670067), 1,1‐diphenyl‐2‐picrylhydrazyl (PubChem CID: 5360881), butylated hydroxytoluene (PubChem CID: 31404), Folin–Ciocalteu (PubChem CID 53384541), nitric acid (PubChem CID: 944), sulfuric acid (PubChem CID: 1118), and aluminum chloride (Pubchem CID: 24012) were used for the characterization of the phytochemical profile and antioxidant activities. In the physiological and biochemical assessments, the following reagents were used: yohimbine (PubChem CID: 8969), loperamide (PubChem CID: 3955), phenol red (PubChem CID: 4766), methyl cellulose (PubChem CID: 51063134), gum Arabic (PubChem CID: 24841), charcoal meal, thiobarbituric acid (PubChem CID: 2723628), trichloroacetic acid (PubChem CID: 6421), reduced glutathione (PubChem CID: 124886), Tris buffer (PubChem CID: 6281), ethylenediaminetetraacetic acid (PubChem CID: 60479), bovine catalase, hydrogen peroxide (PubChem CID: 784), methanol (PubChem CID: 887), eosin (PubChem CID: 34579), and Ketamine (PubChem CID: 3821). All reagents used were of analytical grade and obtained from Sigma Chemical Co. (Sigma‐Aldrich GmbH, Steinheim, Germany).

### 2.2 Plant collection and aqueous extract of beetroot leaves (AEBL) preparation

Beetroot leaves (*Beta vulgaris* L.) were harvested in May 2024 from Béja (Northwest Tunisia). The plant material was identified and authenticated by a taxonomy specialist at the Higher Institute of Biotechnology of Béja, University of Jendouba, and was assigned the identification number BRL‐ISBB‐10/06/24. After collection, the leaves were air‐dried in the shade to minimize nutrient degradation. Once completely dried, they were finely ground using a mechanical grinder to obtain a homogeneous powder. This powder was then stored in airtight containers, protected from air, humidity, light, and biological contaminants such as fungi and insects, to prevent any deterioration.

To prepare the aqueous extract, the powdered leaves were dissolved in bi‐distilled water and incubated for 24 h under continuous agitation in a shaking bath. After incubation, the mixture was centrifuged at 10,000 × *g* for 10 min to eliminate insoluble residues. The supernatant was then lyophilized to obtain a dry extract, which was stored at −20°C until use.

For in vivo administration, fresh working solutions were prepared daily by reconstituting the lyophilized extract in distilled water to the required concentrations and administered orally within 1 h to ensure stability and bioactivity. Routine quality control was conducted for each batch to confirm chemical consistency. Specifically, total polyphenol and flavonoid contents were measured using standardized spectrophotometric methods (the Folin–Ciocalteu and aluminum chloride assays, respectively). These compounds were selected as markers due to their pharmacological relevance and sensitivity to compositional changes. All batches used in vivo showed minimal variation (≤5%) in these parameters, confirming the chemical stability and reproducibility of the extract throughout the study.

### 2.3 Nutritional value of AEBL

#### 2.3.1 Phytochemical screening

The phytochemical composition of beetroot leaves was analyzed to evaluate their bioactive compounds, highlighting their potential therapeutic properties. Total phenolic content was determined using the Folin–Ciocalteu method, following the procedure described by Haseeb et al. (2006). The concentration of flavonoids, known for their significant health benefits, was measured using the aluminum chloride (AlCl_3_) colorimetric method (Chang et al., 2002). Flavonols, another important class of antioxidants, were quantified based on the methodology outlined by Rigane et al. (2011). Additionally, total tannins were assessed using the Folin–Ciocalteu reagent (Kujala et al., 2000), emphasizing their contribution to the leaves' bioactivity. Furthermore, total anthocyanin content, responsible for pigmentation and various health effects, was estimated through an absorbance differentiation method involving two pH buffers: potassium chloride (KCl, pH 1.0, 0.025 M) and sodium acetate (CH_3_COONa, pH 4.5, 0.4 M) (Lee et al., 2005).

#### 2.3.2 Determination of minerals and parietal constituents

The mineral content of AEBL was evaluated following the method described by Jian et al. (2000). A total of 27 mL of the extract was digested with 8 mL of nitric acid (HNO_3_) for 16 h at room temperature. The mixture was then heated in a block digester (ED54 Digiblock Digester Lab Tech, USA) at 133°C until the complete volatilization of the acid. After cooling, 2 mL of distilled water was added, and the mixture was returned to the block digester under the same conditions. Subsequently, 40 mL of distilled water was added, and the solution was filtered through a syringe filter with a 0.4 μm pore diameter. The mineral concentrations in the prepared samples were determined using an atomic absorption spectrophotometer (AVANTA GBC Scientific Equipment Pty. Ltd., Australia).

The cell wall constituents of the plant, such as cellulose, hemicelluloses, and lignin, were quantified using the method described by Van Soest et al. (1991). This involved analyzing the neutral detergent‐insoluble residue (NDF), acid detergent‐insoluble residue (ADF), and the insoluble residue to determine the levels of these cell wall components.

#### 2.3.3 Total sugars and betalains quantification

Total sugar content in the aqueous extract of beetroot leaves (AEBL) was determined following the colorimetric method described by Dubois et al. (1956). In brief, 2 mL of the extract was mixed with 1 mL of 5% phenol solution, followed by the careful addition of 5 mL of concentrated sulfuric acid (H_2_SO_4_) to prevent splashing on the tube walls. The reaction mixture was stirred immediately, resulting in the formation of a stable yellow color. The tubes were incubated in a water bath at 25–30°C for 20 min and then cooled to room temperature (25°C). Absorbance was measured at 485 nm using a spectrophotometer. Sugar concentration was calculated using a standard glucose calibration curve and expressed as grams of glucose per gram of dry matter (g/g DM).

Betalain content, including betacyanins and betaxanthins, was quantified spectrophotometrically according to the method described by Stintzing et al. (2005). Absorbance was recorded at 538 nm for betacyanins and 480 nm for betaxanthins. Concentrations were calculated using their respective molar extinction coefficients and reported as milligrams per gram of dry matter (mg/g DM).

### 2.4 Antioxidant activity of AEBL

#### 2.4.1 2‐diphenyl‐ 1‐ picrylhydrazyl (DPPH ^•^) radical scavenging capacity

The scavenging activity of aqueous extract of beetroot leaves (AEBL) for DPPH^•^ radicals was evaluated following the method described by Jridi et al. (2014). Briefly, 500 μL of the test sample was mixed with 375 μL of 100% ethanol and 125 μL of a 0.02 mM DPPH solution in ethanol. The mixture was vortexed and left to incubate in the dark at room temperature for 60 min. The control sample was prepared in the same manner, with distilled water replacing the test sample. Ascorbic acid was used as a positive standard for comparison. After incubation, the absorbance of the reaction mixture was measured at 517 nm. The DPPH radical scavenging activity was calculated using the following formula:

$$\text{Scavenging activity}\;\left(\%\right)=\frac{\left(\mathrm{AC}-\mathrm{AS}\right)}{\mathrm{AC}}\times 100$$

where AC and AS represent the absorbance values of the control and the sample, respectively. The results were expressed as the half‐maximal inhibitory concentration (IC_50_) in μg/mL.

#### 2.4.2 ABTS ^•+^ radical scavenging activity

The antioxidant capacity of the aqueous extract of beetroot leaves (AEBL) was assessed using the ABTS (2, 2′‐azino‐bis [3‐ethylbenzthiazoline‐6‐sulfonic acid]) method (Siddhuraju, 2006). Briefly, 1 mL of the diluted extract was mixed with 3 mL of a 7 mM ABTS^•+^ (ABTS radical cation) solution and incubated in the dark at room temperature for 60 min. The absorbance of the resulting solution was measured at 734 nm.

The scavenging activity was calculated using the following formula:

$$\text{Scavening activity}\;\left(\%\right)=\left(1-A0/\mathrm{Ab}\right)\times 100$$

where Ab represents the absorbance of the sample, and A0 is the absorbance of the ABTS^•+^ solution at 734 nm. Gallic acid was used as a reference molecule at the same concentration as the test extract.

### 2.5 Animals and housing conditions

Physiological studies were conducted using male *Wistar* rats (weighing 220 ± 20 g, aged 15 weeks) and male *Swiss Albino* mice (weighing 25 ± 5 g, housed 8 per cage). These animals were obtained from the Society of Pharmaceutical Industries of Tunisia (SIPHAT, Tunis, Tunisia). The animals were housed in groups of five per polypropylene cage under controlled environmental conditions, with a 12‐h light/dark cycle and a temperature of 22 ± 2°C and relative humidity of 50%–60%. They had ad libitum access to water and a standard laboratory rodent diet (Badr®, Utique, Tunisia), composed mainly of cereals, vegetable proteins, fats, vitamins, and minerals. All animal care and experimental procedures were approved by the Institutional Animal Care and Use Committee of the National Institute of Health and adhered to the NIH Guidelines for the Care and Use of Laboratory Animals.

### 2.6 Acute toxicity assessment of AEBL in an animal mice model

The acute toxicity of aqueous extract of beetroot leaves (AEBL) was evaluated by oral administration in male *Swiss Albino* mice. A series of escalating doses of AEBL (5, 10, 20, 50, 100, 200, 500, 1000, and 2000 mg/kg, *b.w*.) were prepared and administered to 8 groups of male mice (*n* = 10 per group). All substances were administered once orally by gavage using a stainless‐steel feeding needle adapted to the body weight of each mouse and attached to a calibrated syringe. This method ensured precise and safe delivery of the test solutions. A control group was treated with 10 mL/kg of 0.9% NaCl. Observations were made regarding mortality, mobility, respiratory changes, and appetite. Examinations were performed at 30‐min intervals during the first 6 hs of the initial day, followed by once‐daily assessments for 48 h.

### 2.7 Assessment of gastrointestinal motility

#### 2.7.1 Gastrointestinal transit (GIT)

The charcoal meal test was used to evaluate gastrointestinal transit (GIT) following the method described by Ali and Bashir (1993). A total of 36 rats were randomly assigned to six groups (*n* = 6). Before the experiment, all animals were fasted for 16 h with free access to water. The treatment protocols for each group were as follows:

1. Group 1 (Negative control): received saline solution (0.9% NaCl, 5 mL/kg, *b.w*., p.o.).
2. Group 2 (Loperamide group): received loperamide (LOP; 3 mg/kg, *b.w*., p.o.).
3. Groups 3–5 (AEBL‐treated groups): pretreated with the aqueous extract of beetroot leaves (AEBL) at increasing doses (100, 200, and 400 mg/kg, *b.w*., p.o., respectively).
4. Group 6 (Positive Control): received the standard prokinetic drug yohimbine (YOH, 2 mg/kg, *b.w*., p.o.).

After a 2‐h interval, all rats were given a charcoal meal (10% charcoal in 5% gum Arabic). 30 min later, the rats were anesthetized and sacrificed, and the small intestine was carefully removed from the abdominal cavity. The distance traveled by the charcoal meal from the pylorus was measured to calculate GIT using the formula:

$$\mathrm{GIT}\;\left(\%\right)=\left(\;\frac{\text{Distance traveled}\;\mathrm{by}\;\text{the charcoal meal}\;\left(\mathrm{cm}\right)}{\text{Total length of the small intestine}\;\left(\mathrm{cm}\right)}\;\right)\times 100$$

#### 2.7.2 Gastric emptying (GE)

The gastric emptying rate was conducted independently and was assessed using the red phenol method (Scarpignato et al., 1980). A total of 36 rats were used and assigned to the same treatment groups described in Section 2.7.1. After a 1‐h interval, a test meal (1.5 mL/rat) consisting of 50 mg phenol red in 100 mL aqueous methyl cellulose (1.5%) was orally administered.

To determine the standard (0% gastric emptying), phenol red was recovered from a group of animals sacrificed immediately after the administration of the test meal. The remaining animals were sacrificed 1 h later. The stomach and its contents were homogenized with 100 mL of NaOH (0.1 N). After the suspension was allowed to settle for 1 h at room temperature, 5 mL of the supernatant was combined with 0.5 mL of 20% (w/v) trichloroacetic acid (TCA) and centrifuged at 1800 g for 20 min. Then, 4 mL of NaOH (0.5 N) was added to the supernatant, and the absorbance was measured at 560 nm. The percentage of gastric emptying was calculated using the following formula:

$$\text{Gastric emptying rate}\;\left(\%\right)=\frac{1-\text{absorbance of treated}}{\text{absorbance of standard}}\times 100$$

### 2.8 Induction of constipation: Experimental design

Male *Wistar* rats were randomly divided into six groups (*n* = 6 per group). Constipation was induced in all groups except the negative control by oral administration of loperamide (3 mg/kg, *b.w*.) twice daily at 08:30 and 17:30 for 7 consecutive days. The treatment protocol for each group is summarized in Table 1.

**TABLE 1:** Summary of experimental groups and treatment protocols used in the loperamide‐induced constipation model in male rats over 7 consecutive days.

Groups	Treatments
Group 1: Normal control	0.9% NaCl (5 mL/kg, *b.w*., p.o.) once daily
Group 2: LOP (constipated)	Loperamide (3 mg/kg, *b.w*., p.o.) twice daily
Group 3: LOP + AEBL‐100	Loperamide + AEBL (100 mg/kg, p.o.) once daily (1 h after LOP administration)
Group 4: LOP + AEBL‐200	Loperamide + AEBL (200 mg/kg, *b.w*., p.o.) once daily (1 h after LOP administration)
Group 5: LOP + AEBL‐400	Loperamide + AEBL (400 mg/kg, *b.w*., p.o.) once daily (1 h after LOP administration)
Group 6: LOP + YOH	Loperamide + Yohimbine (2 mg/kg, *b.w*., p.o.) once daily (1 h after LOP administration)

Abbreviations: AEBL, aqueous extract of beetroot leaves; b.w., body weight; LOP, loperamide; p.o., per os (oral administration); YOH, yohimbine.

During the 7‐day experimental protocol of loperamide‐induced constipation, the aqueous beetroot leaf extract (AEBL) was prepared freshly each day to preserve its bioactivity. Multiple independent preparations were used throughout the study, each following the same extraction protocol and subjected to chemical validation as described in section 2.2. The reproducibility of the results was confirmed across these independent batches. The selected doses (100, 200, and 400 mg/kg, *b.w*.) were determined based on preliminary dose‐finding studies, which indicated their efficacy in promoting intestinal motility without causing observable toxicity.

Throughout the experimental period, food and water intake were monitored daily; at the conclusion of the 7‐day protocol, all animals were anesthetized with ketamine (70 mg/kg, *b.w*., intraperitoneally) and euthanized. Blood samples with the addition of EDTA were collected and centrifuged at 3000 × g for 20 min at 4°C to obtain plasma. The colons and intestines were dissected, rinsed with physiological saline, and processed for tissue analysis. To ensure consistency across animals, all colonic mucosal samples were collected from the distal colon (2–3 cm above the rectum), and small intestinal mucosa was sampled from the mid‐jejunum region. This standardization was applied for both histological and biochemical analyses.

The samples were homogenized in phosphate‐buffered saline (PBS) and centrifuged at 3000 × g for 15 min at 4°C. The resulting supernatants and plasma were collected and stored for subsequent biochemical and inflammatory marker assessments.

#### 2.8.1 Fecal parameters assessment

One hour after administering the extract or drug each day, the fecal pellets expelled by the animals were collected. The total number and wet weight of feces were recorded daily for 7 consecutive days. To assess fecal water content, the pellets were dried in a laboratory oven at 70°C for 24 h, following the procedure described by Rtibi et al. (2017). After drying, the fecal dry weight was measured. The water content of the feces was then calculated using the following formula:

$$\text{Fecal water content}\;\left(\%\right)=\frac{\text{fecal}\;\mathrm{wet}\;\text{weight}-\text{fecal}\;\mathrm{dry}\;\text{weight}}{\text{fecal}\;\mathrm{wet}\;\text{weight}}\times 100$$

For each group, the mean values of fecal number and water content were expressed as the average over the 7‐day treatment period.

#### 2.8.2 Oxidative stress biomarkers

MDA levels in the intestinal and colonic mucosa were determined using the double‐heating method (Draper & Hadley, 1990). Tissue homogenates were mixed with a BHT‐TCA solution (1% BHT in 20% TCA) and centrifuged (1000 g, 5 min, 4°C). The supernatant was combined with 0.5 N HCl and 120 mM TBA in 26 mM Tris, heated at 80°C for 10 min, then cooled. The absorbance of the resulting chromophore was measured at 532 nm, and MDA concentration was calculated using an extinction coefficient of 1.56 × 10^5^ M^−1^ cm^−1^.

H_2_O_2_ levels in the intestinal and colonic mucosa were assessed using Dingeon's method (Dingeon et al., 1990). In this assay, H_2_O_2_ reacts with p‐hydroxybenzoic acid and 4‐aminoantipyrine in the presence of phenol and peroxidase, forming a pink quinoneimine complex. The absorbance of this complex was measured at 505 nm.

#### 2.8.3 Assessment of antioxidant enzyme activities

The superoxide dismutase (SOD) activity in the intestinal and colonic mucosa was assessed using the method described by Misra and Fridovich (1972). This method is based on the inhibition of epinephrine auto‐oxidation to adenochrome at pH 10.2. The assay mixture consisted of 10 μL of bovine catalase (0.4 U/mL), sodium carbonate/bicarbonate buffer (62.5 mM, pH 10.2), and 20 μL of epinephrine (5 mg/mL). One unit (U) of SOD is defined as the enzyme required to inhibit the rate of epinephrine auto‐oxidation by 50%. The SOD activity was expressed as U/mg of protein in the intestinal and colonic mucosa.

Catalase (CAT) activity in the intestinal and colonic mucosa was measured by monitoring the reduction of hydrogen peroxide (H_2_O_2_) at 240 nm (Aebi, 1974). The reaction mixture contained 33 mM H_2_O_2_, 50 mM phosphate buffer (pH 7), and 30 μL of intestinal and colonic mucosa tissue extract. CAT activity was calculated as μL H_2_O_2_ consumed per minute per milligram of protein, using the extinction coefficient of H_2_O_2_ (40 mM^−1^ cm^−1^).

Glutathione peroxidase (GPx) activity in the intestinal and colonic mucosa was determined following the method of Flohé and Günzler (1984). The reaction mixture contained 0.2 mL of phosphate buffer (0.1 M, pH 7.4), 0.2 mL of reduced glutathione (GSH, 1 mM), 0.4 mL of H_2_O_2_ (5 mM), and 0.2 mL of intestinal or colonic mucosa homogenate. The mixture was incubated for 1 min at 37°C, and the reaction was stopped by adding 0.5 mL of 5% TCA. After centrifugation at 1500 g for 5 min, 0.5 mL of the supernatant was mixed with 0.5 mL of phosphate buffer and 0.5 mL of DTNB (10 mM). Absorbance was measured at 412 nm. GPx activity was expressed as nmol of GSH consumed per minute per milligram of protein in the intestinal and colonic mucosa, using the extinction coefficient of 1.36 × 10^3^ M^−1^.

#### 2.8.4 Reduced glutathione concentrations

The concentration of reduced glutathione (GSH) was measured using the modified method of Sedlak and Lindsay (1968). In brief, 5 mL of the tissue supernatant was combined with 4 mL of cold distilled water and 1 mL of 50% trichloroacetic acid (TCA). The mixture was vortexed for 10 min and then centrifuged at 1200 g for 15 min. Next, 2 mL of the supernatant was mixed with 4 mL of 0.4 M Tris–HCl buffer (pH 8.9), followed by the addition of 100 μL of 0.01 M 5,5′‐dithiobis‐(2‐nitrobenzoic acid) (DTNB). The absorbance of the resulting solution was measured rapidly at 412 nm, using a blank that contained only the buffer. This procedure allowed the quantification of reduced glutathione levels in the intestinal and colonic mucosa.

#### 2.8.5 Intracellular mediators determination

Calcium (Ca^2+^) and free iron contents in both colonic and intestinal mucosa were determined using established colorimetric methods. For calcium determination, the method described by Stern and Lewis (1957) was employed. In this procedure, calcium ions (Ca^2+^) in the mucosal tissues precipitate as calcium oxalate and form a complex with o‐cresol phthalein. The complex undergoes a color change, which is measured colorimetrically at 570 nm. The resulting absorbance is proportional to the calcium concentration in the colonic and intestinal mucosa samples.

On the other hand, free iron levels in both mucosal types were measured using the ferrozine reagent according to the method by Leardi et al. (1998). Ferrozine reacts with iron that has been released from the transferrin‐iron complex by guanidine acetate and reduced by ascorbic acid. This reaction forms a pink‐colored complex that is detectable at 562 nm. The intensity of the pink color correlates with the amount of free iron in the mucosal tissues.

#### 2.8.6 Assessment of inflammatory status

Plasma levels of C‐reactive protein (CRP) and alkaline phosphatase (ALP) activity were measured using the SELECTRA PRO XL automatic biochemical analyzer along with the corresponding commercial assay kits (ELLSA Tech Group Clinical System SAS, Tunisia). The kits used for the quantification of CRP (Ref: 45027) and ALP (Ref: 13033) were purchased from Biomaghreb (Ariana, Tunisia).

#### 2.8.7 Serum electrolyte variation

The potential protective efficacy of the AEBL against LOP‐induced constipation was assessed by analyzing serum levels of key electrolytes. These measurements were performed using commercial kits (Biomaghreb, Ariana, Tunisia), with the following references: sodium (Ref: 14101), potassium (Ref: 14102), and magnesium (Ref: 14103).

### 2.9 Histological examination

After euthanasia, fragments of colonic tissue (2–3 cm above the rectum) from each experimental group were taken and fixed in 10% paraformaldehyde. The tissues were subjected to dehydration using a graded ethanol series, followed by embedding in paraffin blocks. Sections of 5 μm thickness were then cut, deparaffinized, and rehydrated. For histological examination, the sections were stained with hematoxylin and eosin (H&E) according to standard procedures. Histopathological changes in the colon were evaluated under a Leica DM4000B microscope, and images were captured using a Leica DFC300 FX camera for detailed analysis. Each rat was scored individually, with the histopathological score representing the average of three colonic sections analyzed in a blinded manner by two independent observers. Various parameters were assessed and scored based on the criteria described by Lenoir et al. (2011) including epithelial and glandular destruction, crypt dilation, polymorphonuclear and mononuclear infiltration, edema, detachment of dystrophic epithelium, ulceration, vascular congestion, and the depth of inflammation. The histopathological score for each parameter was assigned on a scale from 0 to 4, with higher scores reflecting more severe damage. The final score for each rat was the sum of the individual parameter scores, providing an overall assessment of colonic injury.

### 2.10 Statistical analysis

All results were expressed as the mean ± standard deviation (SD). One‐way analysis of variance (ANOVA) was employed to compare the differences between groups. Statistical analyses were performed using GraphPad Prism version 8.01 (GraphPad Software Inc., La Jolla, CA, USA). A *p* value of less than 0.05 was considered statistically significant.

## 3 RESULTS

### 3.1 Phytochemical evaluation and nutritional value of AEBL

The phytochemical evaluation of aqueous extract of beetroot leaves (AEBL) was performed to identify key bioactive compounds contributing to its therapeutic potential. The quantitative data on secondary metabolites, minerals, and parietal constituents are summarized in Table 2. The analysis revealed that AEBL is notably rich in polyphenols (129.33 ± 15.94 mg GAE/g DM), flavonoids (29.36 ± 7.36 mg QE/g DM), and total tannins (49.05 ± 6.05 mg TAE/g DM). In contrast, the *Beta vulgaris* leave extract presents a moderate content of parietal fibers including neutral detergent fiber (30.01 ± 4.55% of DM), crude lignin (25.66 ± 4.74% of DM), true cellulose (2.02 ± 0.66% of DM), and hemicelluloses (6.88 ± 1.02% of DM). Furthermore, our investigation reveals that AEBL is rich in mineral matter such as zinc, iron, and magnesium, as well as a moderate quantity of total sugars (55.35 ± 4.15 mg G/g DM).

**TABLE 2:** Phytochemical composition, nutritional value, and IC_50_ values for DPPH and ABTS radical‐scavenging activities of the aqueous extract of beetroot leaves (AEBL).

Parameters	AEBL contents
Total polyphenols (mg GAE/g DM)	129.33 ± 15.94
Flavonoids (mg QE/g DM)	29.36 ± 7.36
Flavonols (mg RE/g DM)	2.08 ± 0.52
Total tannins (mg TAE/g DM)	49.05 ± 6.05
Total anthocyanin content (mg CG/g DM)	8.66 ± 1.07
Total sugar (mg G/ g DM)	55.35 ± 4.15
Betacyanins (mg/L)	90.22 ± 10.33
Betaxanthins (mg/L)	65.95 ± 6.88
Zinc (ppm)	223.87 ± 20.3
Magnesium (ppm)	1008.2 ± 187.47
Iron (ppm)	22.87 ± 2.77
Neutral detergent fiber (% of DM)	30.01 ± 4.55
Crude lignin (% of DM)	25.66 ± 4.74
True raw cellulose (% of DM)	2.02 ± 0.66
Hemicellulose (% of DM)	6.88 ± 1.02
DPPH IC_50_ (μg/mL)	55.67 ± 3.87
ABTS (IC_50_, μg/mL)	68.12 ± 4.45
Butylated hydroxytoluene (IC_50_, μg/mL)	33.87 ± 4.09
Gallic acid (IC_50_, μg/mL)	29.11 ± 0.75

*Note*: Data are expressed as mean ± SEM (*n* = 3).

Abbreviations: CG, cyanidine glucosyl‐3; DM, dry matter; G, glucose equivalent; GAE, gallic acid equivalent; QE, quercetin equivalent; RE, rutin equivalent; SEM: standard error of the mean; TAE, tannic acid equivalent.

Finally, we evaluated the pigmentation levels present in the red beetroot leaves. We showed that the leaves contain significant quantities of betacyanins (90.22 ± 10.33 mg/L) and betaxanthins (65.95 ± 6.88 mg/ L), which contribute to their vibrant color (Table 2).

### 3.2 In vitro antioxidant properties of AEBL

The antioxidant capacity of the aqueous extract of beetroot leaves (AEBL) was assessed using two free radical scavenging assays, such as DPPH and ABTS. The results demonstrated that AEBL exhibited notable antioxidant activity in both assays, with IC_50_ values of 55.67 ± 3.87 μg/mL and 68.12 ± 4.45 μg/mL for DPPH and ABTS, respectively. In comparison, Gallic acid and BHT, used as reference molecules, showed superior antioxidant activity with IC_50_ values of 29.11 ± 0.75 μg/mL and 33.87 ± 4.09 μg/mL, respectively. However, the IC_50_ values for AEBL are relatively close to those of the reference molecules. Nonetheless, the antioxidant capacity of AEBL suggests that it can be a valuable source of natural antioxidants (Table 2).

### 3.3 Acute oral toxicity of AEBL

The acute toxicity study demonstrated that oral administration of increasing doses of AEBL (ranging from 5 to 2000 mg/kg, *b.w*.) did not result in any noticeable changes in behavior, sensory nervous system function, gastrointestinal conditions, or vital signs during the course of the experiment. Additionally, there were no significant alterations in body weight, food, or water intake. No mortality or toxic reactions were observed in any of the treatment groups within 48 h following AEBL administration (data not shown). Based on these findings, the LD_50_ of AEBL is considered to be greater than 2000 mg/kg.

### 3.4 Effect of AEBL and YOH on the variation of food intake, body weight gain, and fecal parameters after LOP‐induced constipation

**FIGURE 1:**
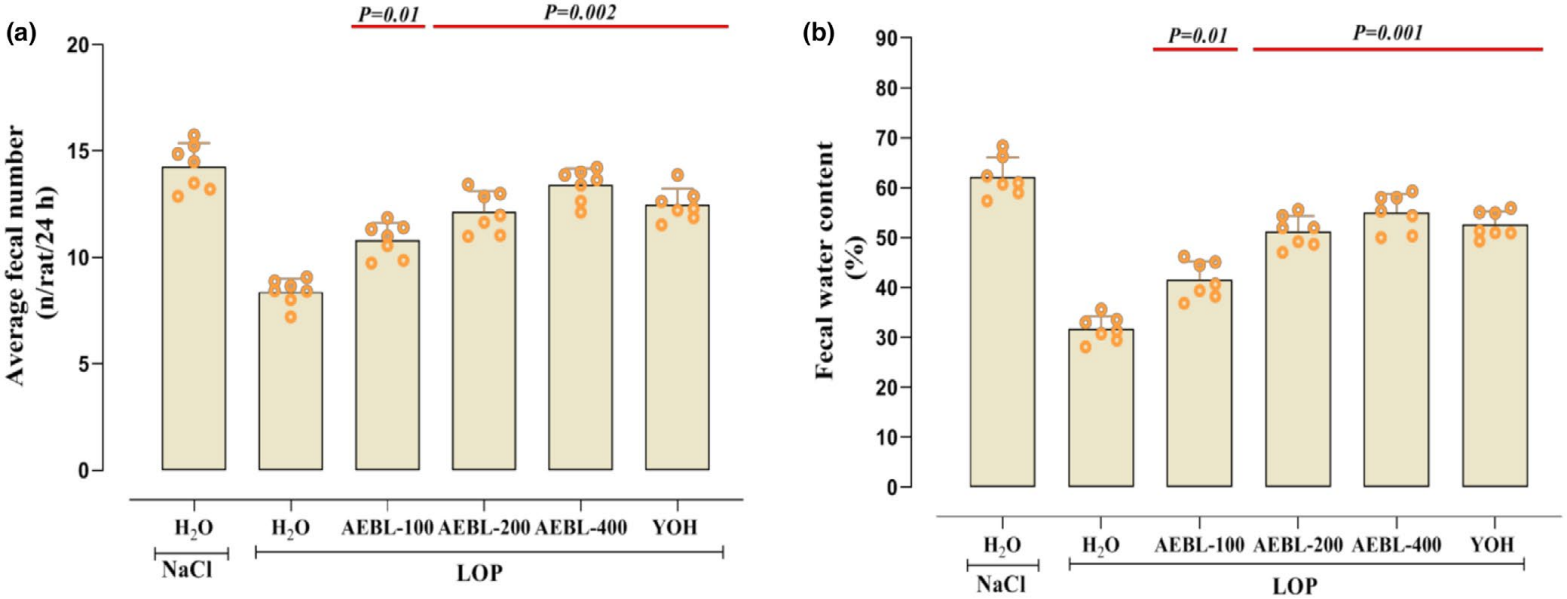
Effects of beetroot leaf aqueous extract (AEBL) and yohimbine (YOH) on Loperamide (LOP)‐induced changes in: (a) Average fecal number and (b) fecal water content (%) recorded during 7‐day treatment. Rats were administered LOP (3 mg/kg, *b.w*., p.o.) to induce constipation and then treated daily with AEBL (100, 200, or 400 mg/kg, *b.w*.) or YOH (2 mg/kg, *b.w*.). Control animals received NaCl (0.9%, 5 mL/kg, *b.w*.) without LOP administration. Data are expressed as mean ± SD (*n* = 7). Statistical differences are indicated by exact *p* values between groups (ANOVA test followed by post hoc test).

After the chemical and antioxidant in vitro evaluation of the aqueous extract of beetroot leaves (AEBL), we proceeded to an in vivo study to assess its therapeutic potential against loperamide (LOP)‐induced constipation. Firstly, our investigation showed that LOP administration (3 mg/kg, *b.w*.) led to a significant reduction in food intake, followed by weight loss in the rats (Table 3). These effects were accompanied by a decrease in fecal output (Figure 1a) and water content (Figure 1b), indicating impaired intestinal motility and fecal characteristics typical of constipation.

**TABLE 3:** Effect of the aqueous extract of beetroot leaves (AEBL, 100, 200, and 400 mg/kg, *b.w*.) and yohimbine (YOH, 2 mg/kg, *b.w*.) on loperamide (LOP, 3 mg/kg, *b.w*.)‐induced changes in food intake and body weight gain in male rats.

Groups	Food intake (g/rat/day)	Body weight gain (g/rat/7 days)
NaCl 0.9%	17.08 ± 2.33	10.22 ± 0.75
LOP	10.87 ± 1.55*	4.57 ± 0.25*
LOP+ AEBL‐100	12.54 ± 0.78^#^	6.87 ± 0.61^#^
LOP + AEBL‐200	14.07 ± 1.10^**#**^	7.55 ± 0.09^#^
LOP + AEBL‐400	15.88 ± 0.32^#^	7.94 ± 0.12^#^
LOP + YOH	15.49 ± 0.22^#^	7.72 ± 0.15^#^

*Note*: Animals in the constipated groups received LOP (3 mg/kg, *b.w*., p.o.) and were subsequently treated with AEBL at 100, 200, or 400 mg/kg, *b.w*. (LOP + AEBL), or with YOH at 2 mg/kg, *b.w*. (LOP + YOH), once daily for 7 days. The control group received NaCl (0.9%, 5 mL/kg, p.o.) without LOP administration. Data are expressed as means ± SD (*n* = 6). *p < 0.05 versus the control group, and #*p* < 0.05 versus the LOP group (ANOVA test).

However, treatment with AEBL, particularly at doses of 200 and 400 mg/kg, resulted in significant improvements in these parameters. A dose‐dependent effect was observed, with the higher doses (400 mg/kg) showing more pronounced effects in restoring food intake, body weight gain, fecal production, and fecal water content. Similarly, yohimbine (2 mg/kg, *b.w*.), used as a reference, also restored these parameters to a level comparable to the control group (Table 3) (Figure 1a,b).

### 3.5 Effect of AEBL and YOH on LOP‐induced gastrointestinal motility deregulation

#### 3.5.1 Effect on gastrointestinal transit rate

**FIGURE 2:**
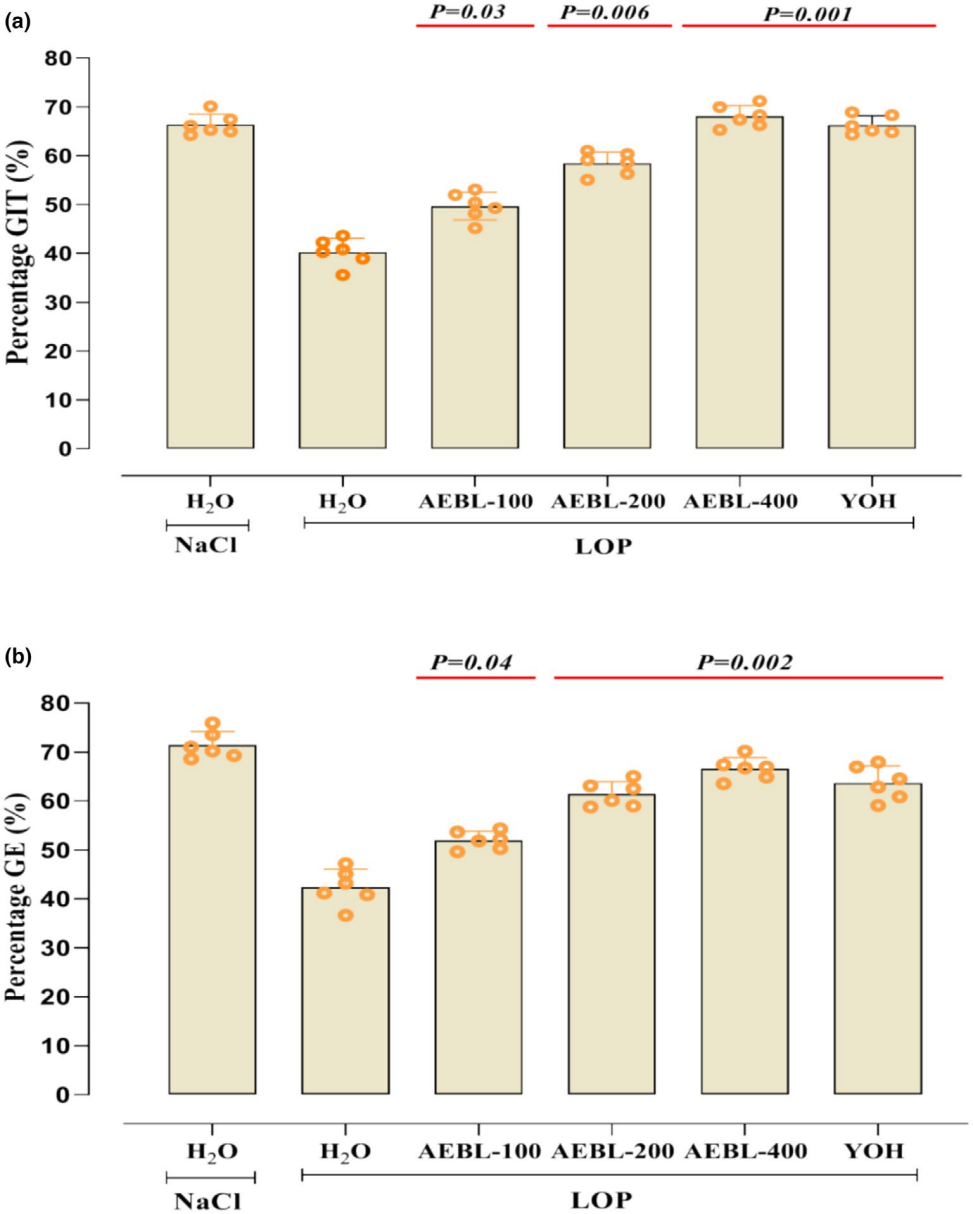
Effect of the aqueous extract of beetroot leaves (AEBL), yohimbine (YOH) and loperamide (LOP) on gastrointestinal motility: (a) Gastrointestinal transit (GIT) assessed in a short‐term experiment. Animals received a single dose of AEBL (100, 200, or 400 mg/kg, *b.w*.), YOH (2 mg/kg, *b.w*.), 0.9% NaCl (5 mL/kg, *b.w*.), or Loperamide (LOP, 3 mg/kg, *b.w*.). After 2 h, all rats were administered a charcoal meal (10% charcoal in 5% gum Arabic, 1 mL/rat) by gavage and sacrificed 30 min later. (b) Gastric emptying (GE) assessed in a separate short‐term experiment. Animals received a single dose of AEBL (100, 200, or 400 mg/kg, *b.w*.), YOH (2 mg/kg, *b.w*.), LOP (3 mg/kg, *b.w*.), or 0.9% NaCl (control). One hour after treatment, all rats were administered by oral gavage a test meal (1.5 mL/rat) containing 50 mg phenol red in 100 mL of 1.5% aqueous methyl cellulose and sacrificed 30 min later. Data are expressed as means ±SD (*n* = 6). Statistical differences are indicated by exact *p* values between groups (ANOVA test followed by post‐hoc test).

In this study, we assessed the effects of AEBL and YOH on gastrointestinal motility in rats subjected to LOP‐induced constipation. The movement of the charcoal meal during the gastrointestinal transit test serves as an indicator of the rate at which food moves through the digestive system, reflecting the frequency of peristaltic contractions. The results from the gastrointestinal transit (GIT) tests are shown in Figure 2a. As anticipated, the LOP group exhibited a notable reduction in the intestinal transit of the charcoal meal (42.87 ± 2.36%) compared to the control group (66.38 ± 3.36%) (*p* < 0.05). However, administration of AEBL at varied doses (100, 200, and 400 mg/kg) resulted in a significant improvement (*p* < 0.05) in small intestinal transit, with the 400 mg/kg dose showing the most substantial effect (69.35 ± 2.56%). These outcomes were similar to those observed in the YOH‐treated group, which also demonstrated a significant increase (*p* < 0.05) in intestinal transit compared to the LOP group.

#### 3.5.2 Effect on gastric emptying

On the other hand, gastric emptying is a crucial process that regulates the movement of nutrients into the upper small intestine. As shown in Figure 2b, a significant (*p* < 0.05) dose‐dependent difference was observed in the amount of phenol red meal emptied in the control group compared to the test groups treated with AEBL or YOH. At a dosage of 400 mg/kg, AEBL increased the emptying of the phenol red meal to 71.72%, a value comparable to that of YOH (65.21%) and the untreated group (68.02%).

### 3.6 Effect of AEBL, YOH, and LOP on the state of oxidative stress in the colonic and intestinal mucosa

#### 3.6.1 Lipoperoxidation and H_2_O_2_ levels

**FIGURE 3:**
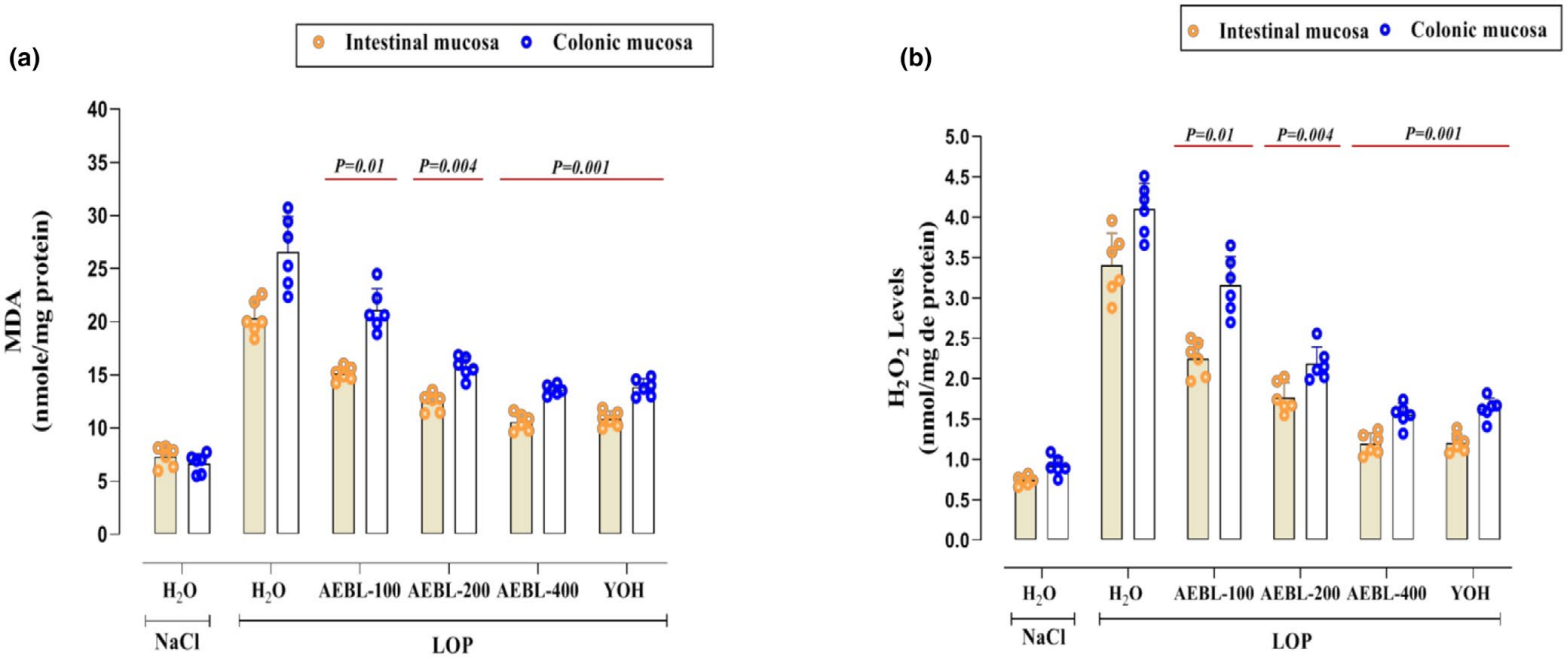
Effect of the aqueous extract of beetroot leaves (AEBL) and yohimbine (YOH) on stress biomarkers: MDA (a), H_2_O_2_ (b) in the colonic and intestinal mucosa during LOP‐induced constipation in rats. Animals in the constipated groups received LOP (3 mg/kg, *b.w*.) and were subsequently treated with various concentrations of AEBL (100, 200, and 400 mg/kg, *b.w*.) or yohimbine (2 mg/kg, *b.w*.), administered 1 h after LOP intoxication, for 7 consecutive days. The control group received NaCl (0.9%, 5 mL/kg, *b.w*.) without LOP treatment. Data are expressed as means ± SD (*n* = 6). Statistical differences are indicated by exact *p* values between groups (ANOVA test followed by post hoc test).

We then proceeded to analyze the oxidative status of the intestine and colon following LOP intoxication in rats. Our results indicate that LOP induces lipid peroxidation, as evidenced by the elevated MDA levels (Figure 3a), along with excessive hydrogen peroxide (H_2_O_2_) production in both the colonic and intestinal mucosa (Figure 3b). However, treatment with increasing doses of *Beta vulgaris* leaves extract (100, 200, and 400 mg/kg) resulted in a significant (*p* < 0.05) and notable reduction in these oxidative stress biomarkers. More importantly, the highest level of protection was observed with the highest dose (400 mg/kg), which exhibited a stronger antioxidant effect compared to the reference laxative molecule (YOH) (Figure 3a,b).

#### 3.6.2 Effect on antioxidant enzyme activity

**FIGURE 4:**
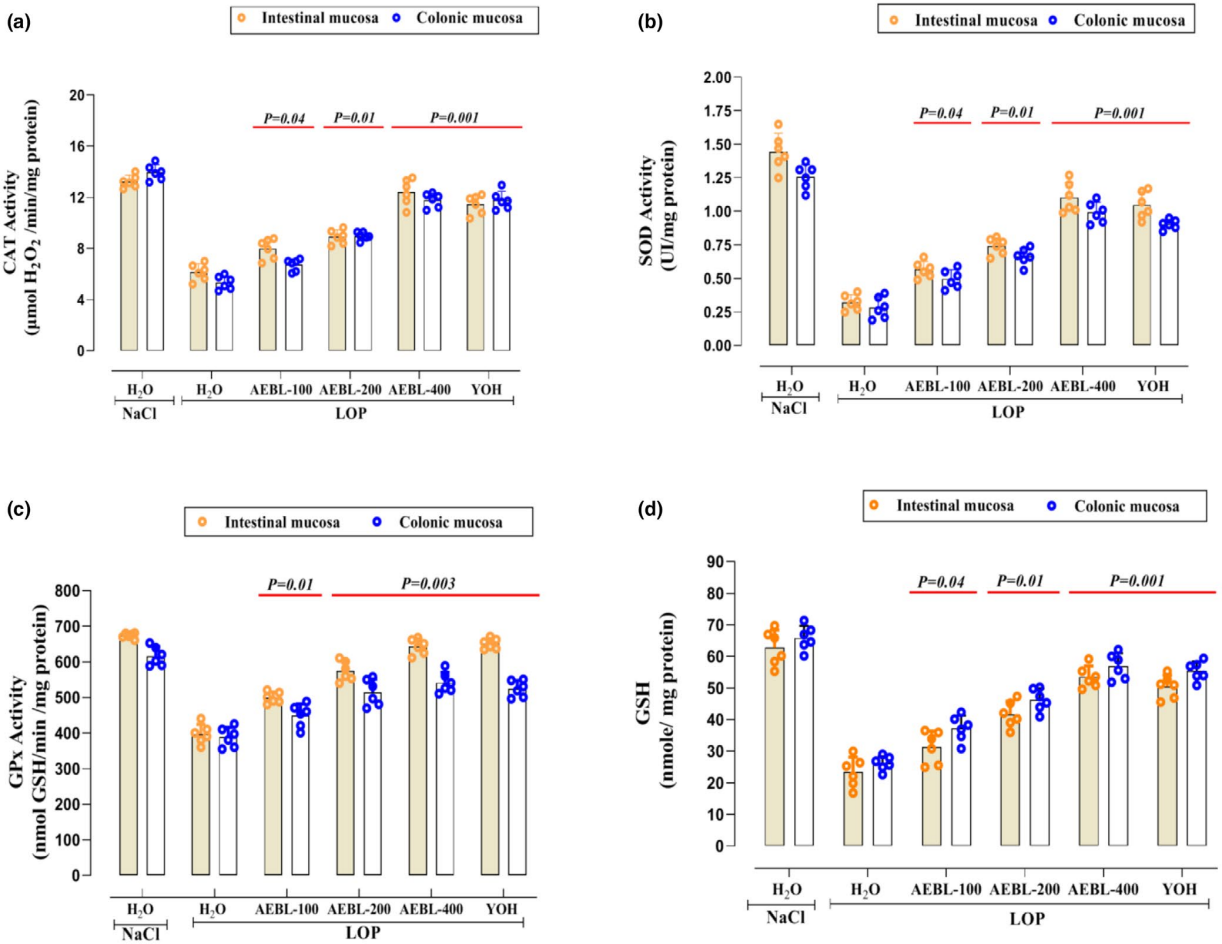
Effect of the aqueous extract of beetroot leaves (AEBL) and yohimbine (YOH) on the antioxidant enzymes activity: CAT (a), SOD (b), GPx (c), and GSH (d) in the colonic and intestinal mucosa during LOP‐induced constipation in rats. Animals in the constipated groups received LOP (3 mg/kg, *b.w*.) and were subsequently treated with various concentrations of AEBL (100, 200, and 400 mg/kg, *b.w*.) or yohimbine (2 mg/kg, *b.w*.), administered 1 h after LOP, for 7 consecutive days. The control group received NaCl (0.9%, 5 mL/kg, *b.w*.) without LOP treatment. Data are expressed as means ± SD (*n* = 6). Statistical differences are indicated by exact *p*‐values between groups (ANOVA test followed by post hoc test).

We assessed the impact of LOP intoxication on the activity of key antioxidant enzymes within the colonic and intestinal mucosa. The results demonstrated a marked decline (*p* < 0.05) in catalase (CAT), superoxide dismutase (SOD), and glutathione peroxidase (GPx) activities (Figure 4a–c, respectively), highlighting a disruption in the antioxidant defense system. Notably, the administration of AEBL at increasing doses (100, 200, and 400 mg/kg) significantly (*p* < 0.05) counteracted this enzymatic suppression, promoting a progressive restoration of antioxidant activity. The highest dose (400 mg/kg) exhibited the greatest efficacy, reinforcing enzymatic defenses to levels comparable to those observed with the standard laxative (YOH). These findings underscore the potent antioxidant properties of AEBL, which contribute to alleviating LOP‐induced oxidative damage.

#### 3.6.3 Effect on Glutathion (GSH) levels

Glutathione (GSH) plays an important role as a major intracellular antioxidant, involved in the detoxification of reactive oxygen species and the maintenance of cellular redox balance. In this study, we assessed the impact of LOP intoxication on GSH levels in both colonic and intestinal mucosa (Figure 4d). Our results demonstrated that LOP administration significantly (*p* < 0.05) decreased GSH levels, suggesting an imbalance in the antioxidant defense system and an increase in oxidative stress. However, treatment with the AEBL at increasing doses (100, 200, and 400 mg/kg) notably (*p* < 0.05) restored GSH levels in a dose‐dependent manner. These findings indicate that AEBL can effectively restore GSH levels, helping to alleviate oxidative damage caused by LOP.

#### 3.6.4 Effect on intracellular mediators

**FIGURE 5:**
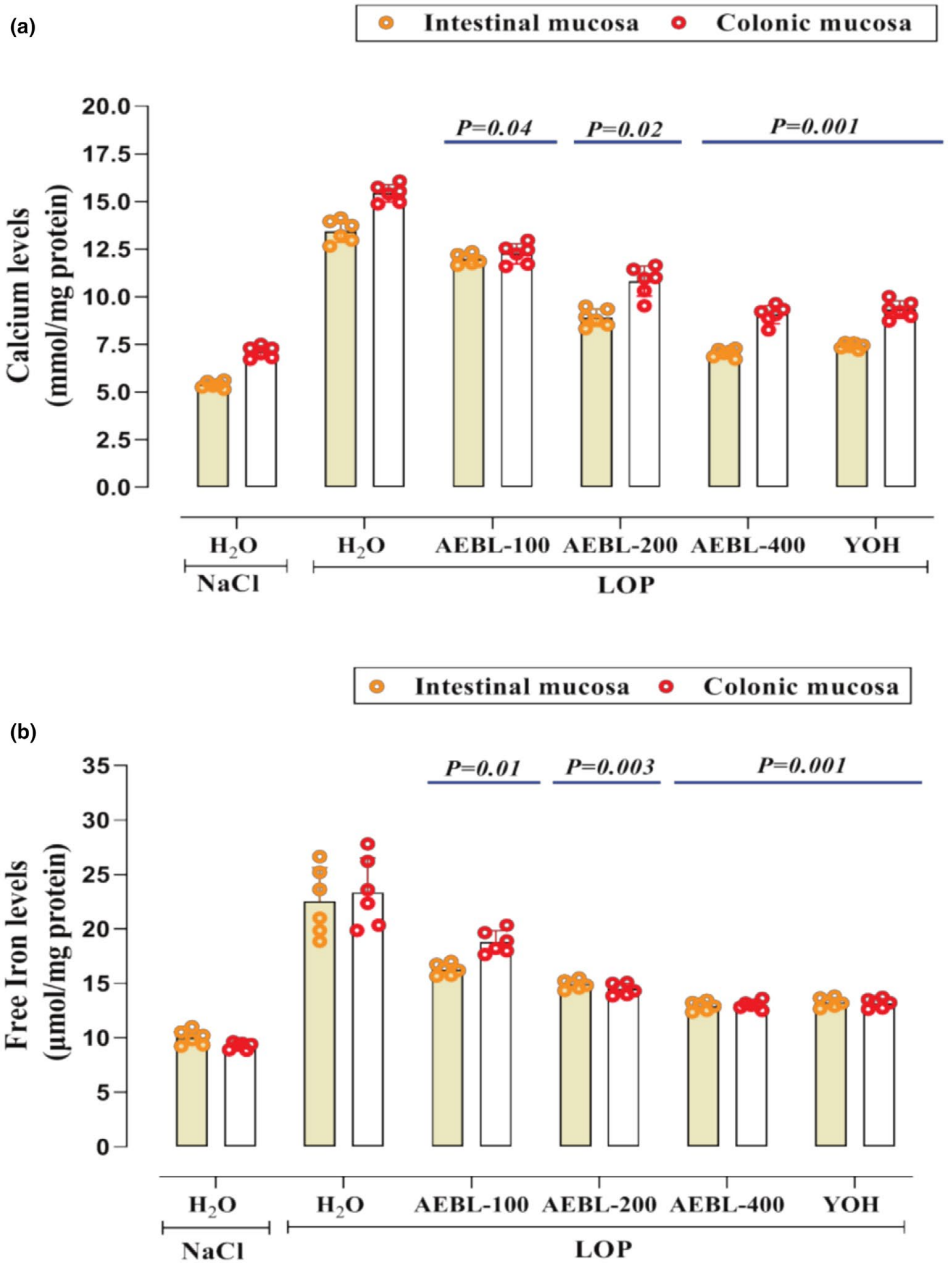
Effect of the aqueous extract of beetroot leaves (AEBL) and yohimbine (YOH) on intracellular mediators: (a) Calcium levels and (b) free iron levels in the colonic and intestinal mucosa during loperamide‐induced constipation in rats. Animals in the constipated groups received LOP (3 mg/kg, *b.w*.) and were subsequently treated with various concentrations of AEBL (100, 200, and 400 mg/kg, *b.w*.) or yohimbine (2 mg/kg, *b.w*.), administered 1 h after LOP, for 7 consecutive days. The control group received NaCl (0.9%, 5 mL/kg, *b.w*.) without LOP treatment. Data are expressed as means ± SD (*n* = 6). Statistical differences are indicated by exact *p* values between groups (ANOVA test followed by post hoc test).

On the other hand, our investigation revealed that LOP, used as an inducing agent for constipation, significantly increased calcium and free iron levels in both intestinal and colonic mucosa compared to the control group (Figure 5a,b, respectively). This elevation suggests an imbalance in intracellular mediators, contributing to oxidative stress and impaired gastrointestinal function. However, treatment with AEBL exerted a significant (*p* < 0.05) reduction in calcium and free iron levels. Notably, this modulation was comparable to that observed in the YOH‐treated group, reinforcing the potential of AEBL in restoring intracellular homeostasis.

### 3.7 Effect of AEBL, YOH, and LOP on plasma inflammatory markers

C‐reactive protein (CRP) and alkaline phosphatase (ALP) are key inflammatory markers commonly used to assess systemic and tissue‐specific inflammation. In this study, LOP intoxication (3 mg/kg, *b.w*.) provoked a significant (*p* < 0.05) increase in plasma CRP and ALP levels compared to the control group (Table 4), indicating an inflammatory response linked to intestinal dysfunction. However, treatment with AEBL at increasing doses significantly reduced these inflammatory markers in a dose‐dependent manner. The highest dose (400 mg/kg) demonstrated the strongest anti‐inflammatory effect, restoring CRP and ALP levels closer to those of the control group and the reference laxative (YOH, 2 mg/kg, *b.w*.), further supporting its potential in alleviating LOP‐induced intestinal inflammation (Table 4).

**TABLE 4:** Effect of the aqueous extract of beetroot leaves (AEBL, 100, 200, and 400 mg/kg) and yohimbine (YOH, 2 mg/kg) on loperamide (LOP, 3 mg/kg)‐induced changes in plasma inflammatory markers: C‐reactive protein (CRP) and alkaline phosphatase (ALP) in male rats.

Groups	CRP (μg/dL)	ALP (UI/L)
NaCl 0.9%	0.42 ± 0.06	60.32 ± 2.36
LOP	1.25 ± 0.23*	178.03 ± 3.21*
LOP + AEBL‐100	1.02 ± 0.15^#^	122.95 ± 4.02^#^
LOP + AEBL‐200	0.71 ± 0.09^#^	90.88 ± 3.94^#^
LOP + AEBL‐400	0.59 ± 0.08^#^	73.21 ± 5.02^#^
LOP + YOH	0.62 ± 0.03^#^	74.98 ± 2.55^#^

*Note*: Animals in the constipated groups received LOP (3 mg/kg, *b.w*., p.o.) and were subsequently treated with AEBL at 100, 200, or 400 mg/kg, *b.w*. (LOP + AEBL), or with YOH at 2 mg/kg, *b.w*. (LOP + YOH), once daily for 7 days. The control group received NaCl (0.9%, 5 mL/kg, p.o.) without LOP administration. Data are expressed as means ± SD (*n* = 6). *p < 0.05 versus the control group, and ^#^ *p* < 0.05 versus the LOP group (ANOVA test).

### 3.8 Effect of AEBL, YOH, and LOP on serum electrolyte level variations

Electrolytes, including magnesium, sodium, and potassium, are essential for maintaining fluid balance, neuromuscular function, and overall cellular homeostasis. In our investigation, LOP administration significantly (*p* < 0.05) disrupted serum electrolyte levels, leading to a marked decrease in magnesium and potassium levels while increasing sodium levels compared to the untreated group. However, treatment with the aqueous extract of beetroot leaves at increasing doses (100, 200, and 400 mg/kg) effectively restored electrolyte balance in a dose‐dependent manner, bringing values closer to those observed in the control and YOH‐treated groups (Table 5).

**TABLE 5:** Effect of aqueous extract of beetroot leaves (AEBL, 100, 200, and 400 mg/kg) and yohimbine (YOH, 2 mg/kg) on loperamide (LOP, 3 mg/kg)‐induced alterations in serum electrolyte levels (magnesium, sodium, and potassium) in male rats.

Groups	Magnesium (mmol/L)	Sodium (mmol/L)	Potassium (mmol/L)
NaCl 0.9%	1.51 ± 0.31	192.22 ± 7.44	8.05 ± 0.82
LOP	2.47 ± 0.40*	119.02 ± 4.33*	15.37 ± 0.91*
LOP + AEBL‐100	2.08 ± 0.31^**#**^	130.00 ± 3.97^**#**^	13.72 ± 0.66^**#**^
LOP + AEBL‐200	1.89 ± 0.33^**#**^	166.07 ± 5.11^**#**^	10.13 ± 0.52^**#**^
LOP + AEBL‐400	1.63 ± 0.22^**#**^	175.28 ± 4.05^**#**^	8.87 ± 0.77^**#**^
LOP + YOH	1.67 ± 0.19^**#**^	170.97 ± 6.23^**#**^	8.90 ± 0.85^**#**^

*Note*: Animals in the constipated groups received LOP (3 mg/kg, *b.w*., p.o.) and were subsequently treated with AEBL at 100, 200, or 400 mg/kg, *b.w*. (LOP + AEBL), or with YOH at 2 mg/kg, *b.w*. (LOP + YOH), once daily for 7 days. The control group received NaCl (0.9%, 5 mL/kg, p.o.) without LOP administration. Data are expressed as means ± SD (*n* = 6). *p < 0.05 versus the control group, and ^#^ *p* < 0.05 versus the LOP group (ANOVA test).

### 3.9 Effect of AEBL, YOH, and LOP on histopathological alterations in colonic architecture

**FIGURE 6:**
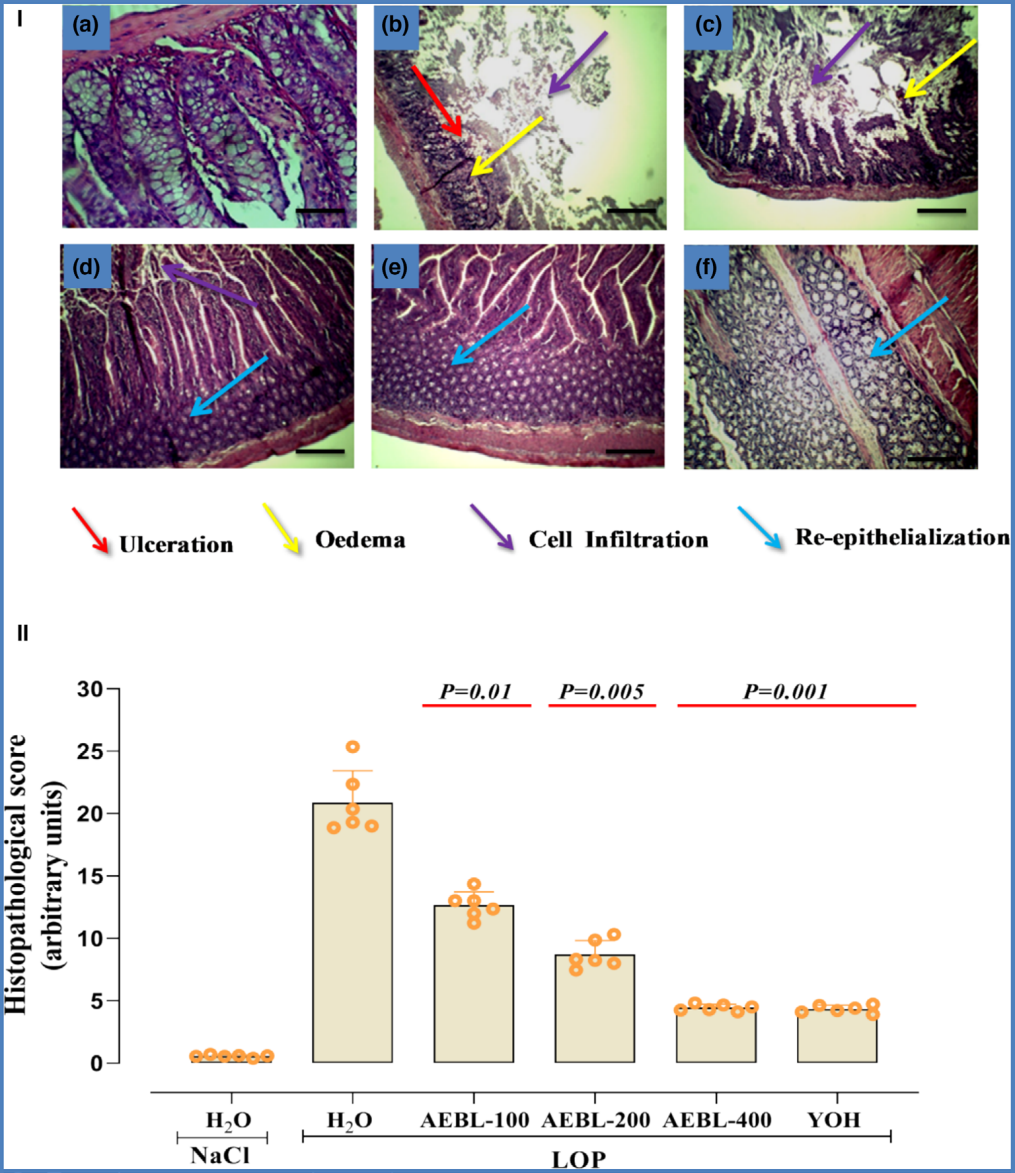
Effect of aqueous extract of beetroot leaves (AEBL) and yohimbine (YOH) on colonic histology (I) and histological score (II) During loperamide (LOP)‐induced constipation in rats. Animals in the constipated groups received LOP (3 mg/kg, *b.w*.) and were subsequently treated with various concentrations of AEBL (100, 200, and 400 mg/kg, *b.w*.) or Yohimbine (2 mg/kg, *b.w*.), 1 h after LOP intoxication, for 7 consecutive days. The control group received NaCl (0.9%, 5 mL/kg, p.o.) without LOP treatment. The groups include: Control (a), LOP (b), AEBL‐100 mg/kg + LOP (c), AEBL‐200 mg/kg + LOP (d), AEBL‐400 mg/kg + LOP (e), and YOH + LOP (f). Histopathological alterations in colon tissue slides were examined using H&E staining, with images captured at ×40 magnification (scale bar = 40 μm). Data are expressed as means ± SD (*n* = 6). Statistical differences are indicated by exact *p* values between groups (ANOVA test followed by post hoc test).

Loperamide intoxication exerted structural alterations in the colonic mucosa, including epithelial damage, inflammatory cell infiltration, and disruption of normal tissue architecture (Figure 6a, LOP group). Our findings indicate that LOP administration resulted in significant histopathological changes, characterized by reduced mucosal integrity and a significantly increased histopathological score (*p* < 0.05) compared to the control group (Figure 6b). However, treatment with AEBL at increasing doses (100, 200, and 400 mg/kg) significantly alleviated these histopathological alterations in a dose‐dependent manner, as evidenced by a progressive reduction in the histopathological score (Figure 6b). The highest dose (400 mg/kg) demonstrated the most pronounced protective effect, restoring colonic architecture, reducing inflammatory infiltration, and promoting mucosal repair (Figure 6a, AEBL‐400 group). Notably, the histopathological score in the AEBL‐400 mg/kg group was significantly lower (*p* < 0.05) than that of the LOP group and approached values observed in the YOH‐treated group, which also exhibited substantial tissue protection. In summary, our results suggest that AEBL can effectively protect the colonic mucosa from LOP‐induced histopathological changes, as reflected by its ability to lower the histopathological score and preserve colonic integrity.

## 4 DISCUSSION

Conventional medications provide essential therapeutic strategies for managing gastrointestinal diseases (Chang & Hanauer, 2017; Laube et al., 2021). However, their prolonged use is often associated with adverse effects such as altered gut motility, dependency, and disruption of the intestinal microbiota (Cha et al., 2023). Consequently, there is growing interest in alternative, plant‐based therapies that offer safer and more sustainable solutions (Gao et al., 2021). Among these, *Beta vulgaris* L., commonly known as red beetroot, has gained scientific attention due to its rich nutritional profile and abundance of bioactive compounds (Hadipour et al., 2020). In this study, we analyzed the phytochemical composition, nutritional value, and the laxative effect of beetroot leaves aqueous extract against loperamide‐induced constipation, oxidative stress, and inflammation in a rat's model.

Firstly, our phytochemical characterization of the aqueous extract of beetroot leaves (AEBL) revealed a diverse profile of bioactive compounds, including polyphenols, flavonoids, tannins, and betalains. These findings are in line with previous research highlighting *Beta vulgaris* L. as a rich source of secondary metabolites with significant biological activities (Barba‐Espin et al., 2018; Giampaoli et al., 2021). Among these compounds, betalains are particularly noteworthy due to their distinct functional properties. These water‐soluble nitrogen‐containing pigments are classified into two main subclasses: betacyanins, responsible for the red‐violet coloration, and betaxanthins, which impart a yellow‐orange hue. Beyond their role as natural colorants, betalains exhibit potent antioxidant activity, acting as free radical scavengers that reduce lipid peroxidation and enhance cellular defense mechanisms (Madadi et al., 2020).

In addition to their strong antioxidant capacity, betalains have been reported to possess anti‐inflammatory, hepatoprotective, and potential anticancer properties, making them valuable bioactive molecules with promising health applications (Martinez et al., 2024). The high betalain content in beetroot leaves, as observed in our study, further reinforces their nutritional and therapeutic significance.

On the other hand, we demonstrated that beetroot leaves are an important source of dietary fiber, sugars, and essential minerals, including calcium, magnesium, potassium, and iron. These compounds highlight the nutritional properties of the plant, contributing to its potential health benefits. Dietary fiber supports digestive health (Makki et al., 2018), while essential minerals play key roles in electrolyte balance, neuromuscular function, and metabolic processes (Sagar et al., 2024). The presence of natural sugars further enhances the energy‐providing potential of beetroot leaves, reinforcing their value as a functional food with broad nutritional applications.

The high content of these bioactive molecules in beetroot leaves suggests that they could serve as a valuable natural source of antioxidants. This was confirmed by in vitro assays using DPPH^•^ and ABTS^•+^ radicals, where the aqueous extract of beetroot leaves exhibited significant free radical‐scavenging activity. The IC_50_ values obtained were comparable to those of standard antioxidant molecules such as ascorbic acid and butylated hydroxytoluene (BHT), indicating the strong antioxidant potential of the extract. Our findings are further supported by our previous study, in which fresh beetroot juice demonstrated similar antioxidant properties, reinforcing the role of *Beta vulgaris* L. as a potent source of natural antioxidants (Ayari, Dakhli, et al., 2025; Ayari, Jedidi, et al., 2025). More importantly, polyphenols and flavonoids, two major classes of bioactive compounds found in beetroot leaves, play a crucial role in free radical neutralization. These secondary metabolites act as potent antioxidants by donating hydrogen atoms or electrons to stabilize reactive oxygen species (Fernando et al., 2016; Ji et al., 2020).

In vivo, we firstly assessed the oral toxicity of the AEBL to determine its safety profile. Our findings showed that oral administration of AEBL at increasing doses (5–2000 mg/kg) did not induce any noticeable behavioral changes, alterations in sensory responses, gastrointestinal disturbances, or fluctuations in vital signs in treated animals. Moreover, there were no significant differences in body weight, food and water intake, or signs of systemic toxicity compared to the control group, suggesting that AEBL is well‐tolerated. Based on these results, the lethal dose (LD_50_) of the extract is estimated to be greater than 2000 mg/kg, indicating its broad margin of safety for potential therapeutic applications.

Furthermore, loperamide‐induced constipation is a widely used experimental model for studying gastrointestinal dysfunction (Gao et al., 2022; Wang et al., 2023). LOP, a peripherally acting opioid receptor agonist, exerts its constipating effect by inhibiting intestinal motility, prolonging transit time, and increasing water absorption in the colon (Baker, 2007). Our investigation confirmed the role of LOP in disrupting gastrointestinal motility, as evidenced by a significant decrease in both gastrointestinal transit and gastric emptying. This impairment led to a marked reduction in the number of fecal pellets and fecal water content, indicating severe constipation. These findings corroborate with previous studies demonstrating that LOP induces constipation through its inhibitory effects on intestinal smooth muscle contraction and fluid secretion (Hajji et al., 2020; Jabri et al., 2017).

However, treatment with the beetroot leaves aqueous extract effectively alleviated these effects in a dose‐dependent manner. Administration of the extract improved gastrointestinal transit and gastric emptying, leading to increased fecal output and water content compared to the constipated group. Notably, at its highest dose, the extract exhibited a motility‐enhancing effect comparable to Yohimbine, a well‐known α2‐adrenergic receptor antagonist that stimulates gut motility through adrenergic modulation (Jabir et al., 2022). These findings suggest that beetroot leaves may support intestinal function, likely due to their fiber content and bioactive compounds, which contribute to enhanced peristalsis and fluid balance in the digestive tract.

More importantly, fiber is known to play a crucial role in gastrointestinal health by modulating intestinal motility and stool consistency (Makki et al., 2018). In the digestive tract, dietary fiber undergoes partial fermentation by gut microbiota, leading to the production of short‐chain fatty acids (acetate, propionate, and butyrate) (Blaak et al., 2020). The presence of soluble fiber in beetroot leaves may contribute to osmotic balance, further improving fecal hydration and easing defecation.

Additionally, tannins, a class of polyphenolic compounds widely found in plant‐based foods, are known for their astringent properties and ability to interact with proteins and other macromolecules (Soares et al., 2020). Their presence in beetroot leaves suggests a potential role in modulating gut function. Tannins can bind to dietary proteins and digestive enzymes, forming complexes that may alter nutrient bioavailability and slow down protein digestion. This interaction not only influences nutrient absorption but also plays a key role in shaping the gut microbiota composition by selectively inhibiting the growth of certain pathogenic bacteria while promoting beneficial microbial populations (Liu et al., 2023).

Moreover, many studies relate oxidative stress to gastrointestinal diseases, highlighting its critical role in the pathogenesis of intestinal dysfunction. Excessive production of reactive oxygen species can disrupt cellular homeostasis, leading to lipid peroxidation, protein oxidation, and DNA damage, which compromise intestinal integrity and function (Popovic et al., 2023; Tomasello et al., 2016). In our study, constipation was associated with increased oxidative stress markers, including elevated lipid peroxidation levels and a decline in enzymatic and non‐enzymatic antioxidant defenses in both colonic and intestinal mucosa. These findings are consistent with previous reports indicating that oxidative imbalance contributes to gut motility disturbances and epithelial barrier dysfunction (Jabri et al., 2017). The administration of AEBL extract significantly restored antioxidant capacity, as evidenced by a reduction in lipid peroxidation and the enhancement of superoxide dismutase, catalase, and glutathione levels. This antioxidant protection may be attributed to the high polyphenol and betalain content of beetroot leaves, which are known for their strong free radical‐scavenging activity. Polyphenols, such as phenolic acids, play a crucial role in neutralizing reactive oxygen species and enhancing endogenous antioxidant defenses (Liu et al., 2019). Betalains, including betacyanins and betaxanthins, further contribute to oxidative stress reduction by stabilizing free radicals and modulating redox balance (Li et al., 2019). Many studies have utilized plant extracts rich in these bioactive compounds to mitigate oxidative stress, including those derived from green tea (Asbaghi et al., 2024), turmeric (Kim et al., 2024), and grape seeds (Silvan et al., 2021).

More importantly, intracellular mediators, such as calcium and free iron, are pivotal in orchestrating cellular responses to oxidative stress, and their dysregulation can exacerbate damage within tissues, particularly in gastrointestinal disorders (Sammari et al., 2021). However, LOP increases calcium and free iron levels in both colonic and intestinal mucosa. Elevated intracellular calcium levels can activate various enzymes, including phospholipases, proteases, and endonucleases, which contribute to membrane disruption, protein degradation, and DNA fragmentation (Farghali & Masek, 1998). Similarly, free iron, particularly in its ferrous state, participates in the Fenton reaction, where it catalyzes the conversion of hydrogen peroxide into highly reactive hydroxyl radicals (Sammari et al., 2021). These hydroxyl radicals can initiate lipid peroxidation, protein oxidation, and DNA damage, further promoting inflammation and tissue injury (Hajji et al., 2020). However, treatment with the aqueous extract of beetroot leaves and YOH significantly mitigated these oxidative effects in a dose‐dependent manner (*p* < 0.05), potentially through their antioxidant properties and modulation of intracellular mediators.

Furthermore, LOP intoxication induced an inflammatory state, as evidenced by elevated levels of C‐reactive protein (CRP). This inflammatory response can be attributed to the activation of several signaling pathways, including cyclooxygenase‐2 (COX‐2) and nuclear factor kappa B (NF‐κB), which are key mediators of inflammation. The activation of COX‐2 leads to the production of pro‐inflammatory prostaglandins, while NF‐κB triggers the expression of various inflammatory cytokines, both contributing to the exacerbation of gastrointestinal inflammation (Desai et al., 2018; Korbecki et al., 2021). In contrast to LOP‐induced inflammation, the aqueous extract of beetroot leaves, rich in anti‐inflammatory agents such as betalains, significantly mitigated the elevated levels of CRP and ALP, highlighting its potent anti‐inflammatory properties. Moreover, the extract's effect was comparable to that of the reference laxative molecule (YOH), known for its ability to enhance gut motility and alleviate inflammation (Ayari, Dakhli, et al., 2025; Ayari, Jedidi, et al., 2025). These results suggest that AEBL could serve as a natural alternative for managing inflammation in gastrointestinal disorders.

Finally, the histopathological analysis revealed significant structural changes in the colonic mucosa after 1 week of LOP administration, notably a significant reduction in mucosal thickness, submucosal edema, and dense infiltration of inflammatory cells. These structural disruptions were accompanied by epithelial degeneration and a notable depletion of goblet cells, which are essential for mucus production and mucosal defense (Yang & Yu, 2021). Such findings are in agreement with earlier studies reporting similar histological damage in LOP‐induced constipation models (Ayari, Dakhli, et al., 2025; Ayari, Jedidi, et al., 2025; Li et al., 2021; Su et al., 2023). Importantly, histological scoring further confirmed the severity of tissue damage in the LOP‐treated group. This loss of epithelial integrity could lead to a weakened mucus barrier, thereby promoting further inflammation and gastrointestinal dysfunction (Su et al., 2023).

In contrast, the present study demonstrated that pretreatment with AEBL at doses of 100, 200, and 400 mg/kg exerted a dose‐dependent histoprotective effect. AEBL effectively preserved mucosal thickness, attenuated inflammatory infiltration, and maintained goblet cell populations, suggesting a strong capacity to counteract LOP‐induced epithelial injury. These protective effects are likely due to the high content of polyphenols, flavonoids, and betalains in AEBL, compounds widely recognized for their antioxidant and anti‐inflammatory actions (Moreno‐Ley et al., 2021; Zhang & Tsao, 2016).

Comparable histoprotective effects have been observed with other plant‐based treatments. For instance, Wahabi et al. (2024) demonstrated that *Arbutus unedo* fruits exerted beneficial laxative and mucosal protective effects in obese constipated rats, while Yakubu and Adams (2025) reported that the aqueous extract of *Cnestis ferruginea* significantly restored normal histoarchitecture in LOP‐induced constipated rats, preserving epithelial structure and reducing inflammation. These comparisons underscore the therapeutic relevance of AEBL and position it as a promising natural intervention for protecting intestinal tissues against Loperamide‐induced colonic injury.

## 5 CONCLUSION

In conclusion, the aqueous extract of beetroot leaves (AEBL) shows promise as a natural remedy for LOP‐induced constipation and gastrointestinal dysfunction. Rich in bioactive compounds like polyphenols, flavonoids, tannins, and betalains, AEBL exhibits strong antioxidant and anti‐inflammatory properties, helping to restore gut balance by neutralizing oxidative stress and regulating intracellular mediators. Additionally, AEBL improved gastrointestinal transit, fecal output, and mucosal integrity. These findings highlight AEBL as a potential alternative to conventional treatments for gastrointestinal disorders.

## AUTHOR CONTRIBUTIONS

Ala Ayari, Saber Jedidi, and Houssem Sammari: Carried out the experiments, analyzed the data, and wrote the article. Nourhène Dhawefi and Soumaya Wahabi: Participated in the processing and analysis of the data. Hichem Sebai: Contributed to the final revision of the manuscript. All authors read and approved the final manuscript.

## FUNDING INFORMATION

The author(s) received no financial support for the research, authorship, and/or publication of this article.

## CONFLICT OF INTEREST STATEMENT

The authors declare that they have no conflicts of interest with respect to their authorship or the publication of this article.

## ETHICS STATEMENT

All animal care and handling procedures were conducted in accordance with the NIH guidelines and the local ethics committee of Tunis University. The protocol was approved by the “Comité d'Éthique Bio‐médicale (CEBM)” (JORT472001) of the “Institut Pasteur de Tunis.”

## Data Availability

The data that support the findings of this study are available upon request from the corresponding author. The data are not publicly available due to privacy or ethical restrictions.

## References

[phy270467-bib-0001] Aebi, H. (1974). Catalase. In H. U. Bergmeyer (Ed.), Methods in enzymatic analysis (pp. 673–686). Verlag Chemie/Academic Press Inc.

[phy270467-bib-0002] Akram, M. , Thiruvengadam, M. , Zainab, R. , Daniyal, M. , Bankole, M. M. , Rebezov, M. , Shariati, M. A. , & Okuskhanova, E. (2022). Herbal medicine for the Management of Laxative Activity. Current Pharmaceutical Biotechnology, 23(10), 1269–1283. 34387161 10.2174/1389201022666210812121328

[phy270467-bib-0003] Ali, B. H. , & Bashir, A. A. (1993). The effect of some alpha 2‐adrenoceptor agonists and antagonists on gastrointestinal transit in mice: Influence of morphine, castor oil and glucose. Clinical and Experimental Pharmacology & Physiology, 20(1), 1–6. 8094327 10.1111/j.1440-1681.1993.tb01495.x

[phy270467-bib-0004] Asbaghi, O. , Rezaei Kelishadi, M. , Larky, D. A. , Bagheri, R. , Amirani, N. , Goudarzi, K. , Kargar, F. , Ghanavati, M. , & Zamani, M. (2024). The effects of green tea extract supplementation on body composition, obesity‐related hormones and oxidative stress markers: A grade‐assessed systematic review and dose‐response meta‐analysis of randomised controlled trials. The British Journal of Nutrition, 131(7), 1125–1157. 38031409 10.1017/S000711452300260X

[phy270467-bib-0005] Ayari, A. , Dakhli, N. , Jedidi, S. , Sammari, H. , Arrari, F. , & Sebai, H. (2025). Laxative and purgative actions of phytoactive compounds from beetroot juice against loperamide‐induced constipation, oxidative stress, and inflammation in rats. Neurogastroenterology and Motility, 37(1), e14935. 39370602 10.1111/nmo.14935

[phy270467-bib-0006] Ayari, A. , Jedidi, S. , Dakhli, N. , Sammari, H. , Dhawefi, N. , & Sebai, H. (2025). Fresh beetroot juice alleviates combined ulcerative colitis and constipation by restoring physiological and biochemical balances in a murine model. Neurogastroenterology and Motility, 2, e70064. 10.1111/nmo.70064 40317625

[phy270467-bib-0007] Aziz, I. , Whitehead, W. E. , Palsson, O. S. , Törnblom, H. , & Simrén, M. (2020). An approach to the diagnosis and management of Rome IV functional disorders of chronic constipation. Expert Review of Gastroenterology & Hepatology, 14(1), 39–46. 31893959 10.1080/17474124.2020.1708718

[phy270467-bib-0008] Baker, D. E. (2007). Loperamide: a pharmacological review. Reviews in Gastroenterological Disorders, 7(Suppl 3), S11–S18. 18192961

[phy270467-bib-0009] Barba‐Espin, G. , Glied‐Olsen, S. , Dzhanfezova, T. , Joernsgaard, B. , Lütken, H. , & Müller, R. (2018). Preharvest application of ethephon and postharvest UV‐B radiation improve quality traits of beetroot (Beta vulgaris L. ssp. vulgaris) as source of colourant. BMC Plant Biology, 18(1), 316. 30509181 10.1186/s12870-018-1556-2 PMC6276243

[phy270467-bib-0010] Blaak, E. E. , Canfora, E. E. , Theis, S. , Frost, G. , Groen, A. K. , Mithieux, G. , Nauta, A. , Scott, K. , Stahl, B. , van Harsselaar, J. , van Tol, R. , Vaughan, E. E. , & Verbeke, K. (2020). Short chain fatty acids in human gut and metabolic health. Beneficial Microbes, 11(5), 411–455. 32865024 10.3920/BM2020.0057

[phy270467-bib-0011] Calvi, P. , Terzo, S. , & Amato, A. (2023). Betalains: Colours for human health. Natural Product Research, 37(10), 1746–1765. 35921318 10.1080/14786419.2022.2106481

[phy270467-bib-0012] Cha, R. R. , Park, S. Y. , Camilleri, M. , & Constipation Research Group of Korean Society of Neurogastroenterology and Motility . (2023). Constipation in patients with chronic kidney disease. Journal of Neurogastroenterology and Motility, 29(4), 428–435. 37814433 10.5056/jnm23133 PMC10577456

[phy270467-bib-0013] Chang, C. , Yang, M. , Wen, H. , & Cher, J. (2002). Estimation of total flavonoid content in propolis by two complementary colorimetric methods. Journal of Food and Drug Analysis, 10, 178–182.

[phy270467-bib-0014] Chang, S. , & Hanauer, S. (2017). Optimizing pharmacologic management of inflammatory bowel disease. Expert Review of Clinical Pharmacology, 10(6), 595–607. 28475384 10.1080/17512433.2017.1318062

[phy270467-bib-0015] Chhikara, N. , Kushwaha, K. , Sharma, P. , Gat, Y. , & Panghal, A. (2019). Bioactive compounds of beetroot and utilization in food processing industry: A critical review. Food Chemistry, 272, 192–200. 30309532 10.1016/j.foodchem.2018.08.022

[phy270467-bib-0016] de Carvalho Nilo Bitu, V. , de Carvalho Nilo Bitu, V. , Matias, E. F. , de Lima, W. P. , da Costa Portelo, A. , Coutinho, H. D. , & de Menezes, I. R. (2015). Ethnopharmacological study of plants sold for therapeutic purposes in public markets in Northeast Brazil. Journal of Ethnopharmacology, 172, 265–272. 26099635 10.1016/j.jep.2015.06.022

[phy270467-bib-0017] De Oliveira, S. P. A. , do Nascimento, H. M. A. , Sampaio, K. B. , & de Souza, E. L. (2021). A review on bioactive compounds of beet (Beta vulgaris L. subsp. vulgaris) with special emphasis on their beneficial effects on gut microbiota and gastrointestinal health. Critical Reviews in Food Science and Nutrition, 61(12), 2022–2033. 32449379 10.1080/10408398.2020.1768510

[phy270467-bib-0018] Desai, S. J. , Prickril, B. , & Rasooly, A. (2018). Mechanisms of phytonutrient modulation of Cyclooxygenase‐2 (COX‐2) and inflammation related to cancer. Nutrition and Cancer, 70(3), 350–375. 29578814 10.1080/01635581.2018.1446091 PMC6309701

[phy270467-bib-0019] Dingeon, B. , Ferry, J. P. , & Roullet, A. (1990). Dosage automatique de la glucosémie par la méthode de Trinder [automatic assay of blood sugar by Trinder's method]. Annales de Biologie Clinique, 33(1), 3–13. 1190573

[phy270467-bib-0020] Draper, H. H. , & Hadley, M. (1990). Malondialdehyde determination as index of lipid peroxidation. Methods in Enzymology, 186, 421–431. 2233309 10.1016/0076-6879(90)86135-i

[phy270467-bib-0021] Dubois, M. , Gilles, K. A. , Hamilton, J. K. , Rebers, P. A. , & Smith, F. (1956). Colorimetric method for determination of sugars and related substances. Analytical Chemistry, 28, 350–356.

[phy270467-bib-0022] Farghali, H. , & Masek, K. (1998). Immunopharmacologic agents in the amelioration of hepatic injuries. International Journal of Immunopharmacology, 20(4–5), 125–139. 9730249 10.1016/s0192-0561(98)00023-x

[phy270467-bib-0023] Fernando, I. P. , Kim, M. , Son, K. T. , Jeong, Y. , & Jeon, Y. J. (2016). Antioxidant activity of marine algal polyphenolic compounds: A mechanistic approach. Journal of Medicinal Food, 19(7), 615–628. 27332715 10.1089/jmf.2016.3706

[phy270467-bib-0024] Flohé, L. , & Günzler, W. A. (1984). Assays of glutathione peroxidase. Methods in Enzymology, 105, 114–121. 6727659 10.1016/s0076-6879(84)05015-1

[phy270467-bib-0025] Gao, C. C. , Li, G. W. , Wang, T. T. , Gao, L. , Wang, F. F. , Shang, H. W. , Yang, Z. J. , Guo, Y. X. , Wang, B. Y. , & Xu, J. D. (2021). Rhubarb extract relieves constipation by stimulating mucus production in the colon and altering the intestinal flora. Biomedicine & Pharmacotherapy = Biomedecine & Pharmacotherapie, 138, 111479. 33774313 10.1016/j.biopha.2021.111479

[phy270467-bib-0026] Gao, X. , Hu, Y. , Tao, Y. , Liu, S. , Chen, H. , Li, J. , Zhao, Y. , Sheng, J. , Tian, Y. , & Fan, Y. (2022). Stapf aqueous extract ameliorates loperamide‐induced constipation in mice by promoting gastrointestinal motility and regulating the gut microbiota. Frontiers in Microbiology, 13, 1017804. 36267178 10.3389/fmicb.2022.1017804 PMC9578511

[phy270467-bib-0027] Giampaoli, O. , Sciubba, F. , Conta, G. , Capuani, G. , Tomassini, A. , Giorgi, G. , Brasili, E. , Aureli, W. , & Miccheli, A. (2021). Red Beetroot's NMR‐based metabolomics: Phytochemical profile related to development time and production year. Foods (Basel, Switzerland), 10(8), 1887. 34441664 10.3390/foods10081887 PMC8393249

[phy270467-bib-0028] Greenwood‐Van Meerveld, B. , Johnson, A. C. , & Grundy, D. (2017). Gastrointestinal physiology and function. Handbook of Experimental Pharmacology, 239, 1–16. 28176047 10.1007/164_2016_118

[phy270467-bib-0029] Hadipour, E. , Taleghani, A. , Tayarani‐Najaran, N. , & Tayarani‐Najaran, Z. (2020). Biological effects of red beetroot and betalains: A review. Phytotherapy Research: PTR, 34(8), 1847–1867. 32171042 10.1002/ptr.6653

[phy270467-bib-0030] Hajji, N. , Wannes, D. , Jabri, M. A. , Rtibi, K. , Tounsi, H. , Abdellaoui, A. , & Sebai, H. (2020). Purgative/laxative actions of globularia alypum aqueous extract on gastrointestinal‐physiological function and against loperamide‐induced constipation coupled to oxidative stress and inflammation in rats. Neurogastroenterology and Motility, 32(8), e13858. 32337785 10.1111/nmo.13858

[phy270467-bib-0031] Haseeb, N. , John, S. , Gauri, M. , & Yukio, K. (2006). Extraction of polyphenols from grape seeds and concentration by ultrafiltration. Separation and Purification Technology, 48, 176–181.

[phy270467-bib-0032] Huang, W. R. , Fang, Q. H. , Yu, X. B. , Ge, W. H. , & Yu, Y. (2024). The potential application of aloe Barbadensis mill. as Chinese medicine for constipation: Mini‐review. Drug Design, Development and Therapy, 18, 307–324. 38328440 10.2147/DDDT.S446563 PMC10849880

[phy270467-bib-0033] Jabir, N. R. , Firoz, C. K. , Zughaibi, T. A. , Alsaadi, M. A. , Abuzenadah, A. M. , Al‐Asmari, A. I. , Alsaieedi, A. , Ahmed, B. A. , Ramu, A. K. , & Tabrez, S. (2022). A literature perspective on the pharmacological applications of yohimbine. Annals of Medicine, 54(1), 2861–2875. 36263866 10.1080/07853890.2022.2131330 PMC9590431

[phy270467-bib-0034] Jabri, M. A. , Wannes, D. , Hajji, N. , Sakly, M. , Marzouki, L. , & Sebai, H. (2017). Role of laxative and antioxidant properties of Malva sylvestris leaves in constipation treatment. Biomedicine & Pharmacotherapy = Biomedecine & Pharmacotherapie, 89, 29–35. 28214685 10.1016/j.biopha.2017.02.020

[phy270467-bib-0035] Ji, M. , Gong, X. , Li, X. , Wang, C. , & Li, M. (2020). Advanced research on the antioxidant activity and mechanism of polyphenols from *Hippophae* species–A review. Molecules (Basel, Switzerland), 25(4), 917. 32092874 10.3390/molecules25040917 PMC7071004

[phy270467-bib-0036] Jian, L. , Goessler, W. , & Irgolic, K. J. (2000). Mercury determination with ICP‐MS: Signal suppression by acids. Fresenius' Journal of Analytical Chemistry, 366(1), 48–53. 11225815 10.1007/s002160050010

[phy270467-bib-0037] Jridi, M. , Hajji, S. , Ayed, H. B. , Lassoued, I. , Mbarek, A. , Kammoun, M. , Souissi, N. , & Nasri, M. (2014). Physical, structural, antioxidant and antimicrobial properties of gelatin‐chitosan composite edible films. International Journal of Biological Macromolecules, 67, 373–379. 24709012 10.1016/j.ijbiomac.2014.03.054

[phy270467-bib-0038] Kim, J. W. , Jeong, J. S. , Kim, J. H. , Kim, C. Y. , Chung, E. H. , Ko, J. W. , & Kim, T. W. (2024). Turmeric extract alleviates airway inflammation via oxidative stress‐driven MAPKs/MMPs pathway. International Immunopharmacology, 141, 113018. 39216235 10.1016/j.intimp.2024.113018

[phy270467-bib-0039] Korbecki, J. , Simińska, D. , Gąssowska‐Dobrowolska, M. , Listos, J. , Gutowska, I. , Chlubek, D. , & Baranowska‐Bosiacka, I. (2021). Chronic and cycling hypoxia: Drivers of cancer chronic inflammation through HIF‐1 and NF‐κB activation: A review of the molecular mechanisms. International Journal of Molecular Sciences, 22(19), 10701. 34639040 10.3390/ijms221910701 PMC8509318

[phy270467-bib-0040] Kujala, T. S. , Loponen, J. M. , Klika, K. D. , & Pihlaja, K. (2000). Phenolics and betacyanins in red beetroot (Beta vulgaris) root: Distribution and effect of cold storage on the content of total phenolics and three individual compounds. Journal of Agricultural and Food Chemistry, 48(11), 5338–5342. 11087483 10.1021/jf000523q

[phy270467-bib-0041] Laube, R. , Paramsothy, S. , & Leong, R. W. (2021). Use of medications during pregnancy and breastfeeding for Crohn's disease and ulcerative colitis. Expert Opinion on Drug Safety, 20(3), 275–292. 33412078 10.1080/14740338.2021.1873948

[phy270467-bib-0042] Leardi, A. , Caraglia, M. , Selleri, C. , Pepe, S. , Pizzi, C. , Notaro, R. , Fabbrocini, A. , De Lorenzo, S. , Musicò, M. , Abbruzzese, A. , Bianco, A. R. , & Tagliaferri, P. (1998). Desferioxamine increases iron depletion and apoptosis induced by ara‐C of human myeloid leukaemic cells. British Journal of Haematology, 102(3), 746–752. 9722302 10.1046/j.1365-2141.1998.00834.x

[phy270467-bib-0043] Lee, J. , Durst, R. W. , & Wrolstad, R. E. (2005). Determination of total monomeric anthocyanin pigment content of fruit juices, beverages, natural colorants, and wines by the pH differential method: Collaborative study. Journal of AOAC International, 88(5), 1269–1278. 16385975

[phy270467-bib-0044] Lenoir, L. , Rossary, A. , Joubert‐Zakeyh, J. , Vergnaud‐Gauduchon, J. , Farges, M. C. , Fraisse, D. , Texier, O. , Lamaison, J. L. , Vasson, M. P. , & Felgines, C. (2011). Lemon verbena infusion consumption attenuates oxidative stress in dextran sulfate sodium‐induced colitis in the rat. Digestive Diseases and Sciences, 56(12), 3534–3545. 21688009 10.1007/s10620-011-1784-x

[phy270467-bib-0045] Li, G. , Meng, X. , Zhu, M. , & Li, Z. (2019). Research Progress of Betalain in response to adverse stresses and evolutionary relationship compared with anthocyanin. Molecules (Basel, Switzerland), 24(17), 3078. 31450587 10.3390/molecules24173078 PMC6749444

[phy270467-bib-0046] Li, T. , Hu, M. , Jiang, C. , Zhang, D. , Gao, M. , Xia, J. , Miao, M. , Shi, G. , Li, H. , Zhang, J. , & Yin, Z. (2021). Laxative effect and mechanism of Tiantian capsule on loperamide‐induced constipation in rats. Journal of Ethnopharmacology, 266, 113411. 32980482 10.1016/j.jep.2020.113411

[phy270467-bib-0047] Liu, S. , Wang, K. , Lin, S. , Zhang, Z. , Cheng, M. , Hu, S. , Hu, H. , Xiang, J. , Chen, F. , Li, G. , & Si, H. (2023). Comparison of the effects between tannins extracted from different natural plants on growth performance, antioxidant capacity, immunity, and intestinal Flora of broiler chickens. Antioxidants (Basel), 12(2), 441. 36829999 10.3390/antiox12020441 PMC9952188

[phy270467-bib-0048] Liu, T. H. , Chen, G. L. , Lin, C. H. , Tsai, T. Y. , & Cheng, M. C. (2025). *Lactobacillus plantarum* TWK10 relieves loperamide‐induced constipation in rats fed a high‐fat diet *via* modulating enteric neurotransmitters, short‐chain fatty acids and gut microbiota. Food & Function, 16(1), 181–194. 39641806 10.1039/d4fo02270j

[phy270467-bib-0049] Liu, Y. , Qi, Y. , Chen, X. , He, H. , Liu, Z. , Zhang, Z. , Ren, Y. , & Ren, X. (2019). Phenolic compounds and antioxidant activity in red‐ and in green‐fleshed kiwifruits. Food Research International, 116, 291–301. 30716948 10.1016/j.foodres.2018.08.038

[phy270467-bib-0050] Madadi, E. , Mazloum‐Ravasan, S. , Yu, J. S. , Ha, J. W. , Hamishehkar, H. , & Kim, K. H. (2020). Therapeutic application of Betalains: A review. Plants (Basel), 9(9), 1219. 32957510 10.3390/plants9091219 PMC7569795

[phy270467-bib-0051] Makki, K. , Deehan, E. C. , Walter, J. , & Bäckhed, F. (2018). The impact of dietary fiber on gut microbiota in host health and disease. Cell Host & Microbe, 23(6), 705–715. 29902436 10.1016/j.chom.2018.05.012

[phy270467-bib-0052] Martinez, R. M. , Melo, C. P. B. , Pinto, I. C. , Mendes‐Pierotti, S. , Vignoli, J. A. , Verri, W. A. , & Casagrande, R. (2024). Betalains: A narrative review on pharmacological mechanisms supporting the nutraceutical potential towards health benefits. Foods (Basel, Switzerland), 13(23), 3909. 39682981 10.3390/foods13233909 PMC11640225

[phy270467-bib-0053] Misra, H. P. , & Fridovich, I. (1972). The role of superoxide anion in the autoxidation of epinephrine and a simple assay for superoxide dismutase. The Journal of Biological Chemistry, 247(10), 3170–3175. 4623845

[phy270467-bib-0054] Moreno‐Ley, C. M. , Osorio‐Revilla, G. , Hernández‐Martínez, D. M. , Ramos‐Monroy, O. A. , & Gallardo‐Velázquez, T. (2021). Anti‐inflammatory activity of betalains: A comprehensive review. Human Nutrition & Metabolism, 25, 200126.

[phy270467-bib-0055] Moulick, S. P. , Jahan, F. , Islam, M. B. , Bashera, M. A. , Hasan, M. S. , Islam, M. J. , Ahmed, S. , Karmakar, D. , Ahmed, F. , Saha, T. , Dey, S. S. , Boby, F. , Saha, M. , Saha, B. K. , & Bhuiyan, M. N. H. (2023). Nutritional characteristics and antiradical activity of turmeric (*Curcuma longa* L.), beetroot (*Beta vulgaris* L.), and carrot (*Daucus carota* L.) grown in Bangladesh. Heliyon, 9(11), e21495. 38027870 10.1016/j.heliyon.2023.e21495 PMC10651453

[phy270467-bib-0056] Paknejad, M. S. , Motaharifard, M. S. , Barimani, S. , Kabiri, P. , & Karimi, M. (2019). Traditional, complementary and alternative medicine in children constipation: A systematic review. Daru: Journal of Faculty of Pharmacy, Tehran University of Medical Sciences, 27(2), 811–826. 31734825 10.1007/s40199-019-00297-w PMC6895286

[phy270467-bib-0057] Pergolizzi, J. V., Jr. , Raffa, R. B. , Pappagallo, M. , Fleischer, C. , Pergolizzi, J. , Zampogna, G. , Duval, E. , Hishmeh, J. , LeQuang, J. A. , & Taylor, R., Jr. (2017). Peripherally acting μ‐opioid receptor antagonists as treatment options for constipation in noncancer pain patients on chronic opioid therapy. Patient Preference and Adherence, 11, 107–119. 28176913 10.2147/PPA.S78042 PMC5261842

[phy270467-bib-0058] Popovic, D. , Stojanovic, M. , Milosavljevic, T. , Stojkovic‐Lalosevic, M. , Glisic, T. , Savic, P. , & Filipovic, B. (2023). Oxidative stress in gastrointestinal ulcer disease: A Gastroenterologist's view. Journal of Gastrointestinal and Liver Diseases: JGLD, 32(3), 277–282. 37774208 10.15403/jgld-5172

[phy270467-bib-0059] Punia Bangar, S. , Sharma, N. , Sanwal, N. , Lorenzo, J. M. , & Sahu, J. K. (2022). Bioactive potential of beetroot (Beta vulgaris). Food Research International, 158, 111556. 35840248 10.1016/j.foodres.2022.111556

[phy270467-bib-0060] Rahimi, P. , Abedimanesh, S. , Mesbah‐Namin, S. A. , & Ostadrahimi, A. (2019). Betalains, the nature‐inspired pigments, in health and diseases. Critical Reviews in Food Science and Nutrition, 59(18), 2949–2978. 29846082 10.1080/10408398.2018.1479830

[phy270467-bib-0061] Rehman, S. , Shah, S. , Mehmood Butt, A. , Masood Shah, S. , Jabeen, Z. , & Nadeem, A. (2021). Biochemical profiling and elucidation of biological activities of *Beta vulgaris* L. leaves and roots extracts. Saudi Journal of Biological Sciences, 28(1), 592–602. 33424345 10.1016/j.sjbs.2020.10.048 PMC7785445

[phy270467-bib-0062] Rigane, G. , Ben Salem, R. , Sayadi, S. , & Bouaziz, M. (2011). Phenolic composition, isolation, and structure of a new deoxyloganic acid derivative from Dhokar and Gemri‐Dhokar olive cultivars. Journal of Food Science, 76(7), C965–C973. 21806611 10.1111/j.1750-3841.2011.02290.x

[phy270467-bib-0063] Rtibi, K. , Grami, D. , Selmi, S. , Amri, M. , Sebai, H. , & Marzouki, L. (2017). Vinblastine, an anticancer drug, causes constipation and oxidative stress as well as others disruptions in intestinal tract in rat. Toxicology Reports, 4, 221–225. 28959642 10.1016/j.toxrep.2017.04.006 PMC5615122

[phy270467-bib-0064] Sadowska‐Bartosz, I. , & Bartosz, G. (2021). Biological properties and applications of Betalains. Molecules, 26(9), 2520. 33925891 10.3390/molecules26092520 PMC8123435

[phy270467-bib-0065] Sagar, A. N. , Kalburgi, V. , Vagha, J. D. , Taksande, A. , Meshram, R. J. , & Lohiya, S. (2024). A comprehensive review of the role of magnesium in critical care pediatrics: Mechanisms, clinical impact, and therapeutic strategies. Cureus, 16(8), e66643. 39258079 10.7759/cureus.66643 PMC11386945

[phy270467-bib-0066] Sammari, H. , Jedidi, S. , Selmi, H. , Rtibi, K. , Jabri, M. A. , Jridi, M. , Zouari, N. , Toumi, L. , & Sebai, H. (2021). Protective effects of Crataegus azarolus L. berries aqueous extract against castor oil‐induced diarrhea, oxidative stress, and inflammation in rat. Neurogastroenterology and Motility, 33(6), e14065. 33320393 10.1111/nmo.14065

[phy270467-bib-0067] Scarpignato, C. , Capovilla, T. , & Bertaccini, G. (1980). Action of caerulein on gastric emptying of the conscious rat. Archives Internationales de Pharmacodynamie et de Thérapie, 246(2), 286–294. 7436633

[phy270467-bib-0068] Sedlak, J. , & Lindsay, R. H. (1968). Estimation of total, protein‐bound, and nonprotein sulfhydryl groups in tissue with Ellman's reagent. Analytical Biochemistry, 25(1), 192–205. 4973948 10.1016/0003-2697(68)90092-4

[phy270467-bib-0069] Siddhuraju, P. (2006). The antioxidant activity and free radicalscavenging capacity of phenolics of raw and dry heated moth bean (Vigna aconitifolia) (Jacq.) Marechal seed extracts. Food Chemistry, 99(1), 149–157.

[phy270467-bib-0070] Silvan, J. M. , Gutierrez‐Docio, A. , Guerrero‐Hurtado, E. , Domingo‐Serrano, L. , Blanco‐Suarez, A. , Prodanov, M. , Alarcon‐Cavero, T. , & Martinez‐Rodriguez, A. J. (2021). Pre‐treatment with grape seed extract reduces inflammatory response and oxidative stress induced by *helicobacter pylori* infection in human gastric epithelial cells. Antioxidants (Basel, Switzerland), 10(6), 943. 34208004 10.3390/antiox10060943 PMC8230724

[phy270467-bib-0071] Soares, S. , Brandão, E. , Guerreiro, C. , Soares, S. , Mateus, N. , & de Freitas, V. (2020). Tannins in food: Insights into the molecular perception of astringency and bitter taste. Molecules (Basel, Switzerland), 25(11), 2590. 32498458 10.3390/molecules25112590 PMC7321337

[phy270467-bib-0072] Stern, J. , & Lewis, W. H. (1957). The colorimetric estimation of calcium in serum with ocresolphthalein complexone. Clinica Chimica Acta; International Journal of Clinical Chemistry, 2(6), 576–580. 13500593 10.1016/0009-8981(57)90063-3

[phy270467-bib-0073] Stintzing, F. C. , Herbach, K. M. , Mosshammer, M. R. , Carle, R. , Yi, W. , Sellappan, S. , Akoh, C. C. , Bunch, R. , & Felker, P. (2005). Color, betalain pattern, and antioxidant properties of cactus pear (Opuntia spp.) clones. Journal of Agricultural and Food Chemistry, 53(2), 442–451. 15656686 10.1021/jf048751y

[phy270467-bib-0074] Su, Y. , Zhu, R. , Pang, C. , He, Z. , Wu, B. , & Wang, X. (2023). Laxative effect of Wenyang Yiqi decoction on loperamide‐induced astriction model mice. Annals of Translational Medicine, 11(4), 170. 36923099 10.21037/atm-23-6 PMC10009577

[phy270467-bib-0075] Thiruvengadam, M. , Chung, I. M. , Samynathan, R. , Chandar, S. R. H. , Venkidasamy, B. , Sarkar, T. , Rebezov, M. , Gorelik, O. , Shariati, M. A. , & Simal‐Gandara, J. (2024). A comprehensive review of beetroot (*Beta vulgaris* L.) bioactive components in the food and pharmaceutical industries. Critical Reviews in Food Science and Nutrition, 64(3), 708–739. 35972148 10.1080/10408398.2022.2108367

[phy270467-bib-0076] Tomasello, G. , Mazzola, M. , Leone, A. , Sinagra, E. , Zummo, G. , Farina, F. , Damiani, P. , Cappello, F. , Gerges Geagea, A. , Jurjus, A. , Bou Assi, T. , Messina, M. , & Carini, F. (2016). Nutrition, oxidative stress and intestinal dysbiosis: Influence of diet on gut microbiota in inflammatory bowel diseases. Biomedical Papers of the Medical Faculty of the University Palacky, Olomouc, Czechoslovakia, 160(4), 461–466. 27812084 10.5507/bp.2016.052

[phy270467-bib-0077] Van Soest, P. J. , Robertson, J. B. , & Lewis, B. A. (1991). Methods for dietary fiber, neutral detergent fiber, and nonstarch polysaccharides in relation to animal nutrition. Journal of Dairy Science, 74(10), 3583–3597. 1660498 10.3168/jds.S0022-0302(91)78551-2

[phy270467-bib-0078] Wahabi, S. , Rtibi, K. , Brinsi, C. , Jridi, M. , & Sebai, H. (2024). Overweight/bowel dysmotility crosslinking and analogous laxative actions of two edible wild fruits in obese/constipated rats. Neurogastroenterology and Motility, 36(12), e14933. 39344995 10.1111/nmo.14933

[phy270467-bib-0079] Wang, L. , Wang, J. , Wang, J. , Guo, Z. , Li, Z. , Qiu, J. , & Wang, L. (2024). Soluble and insoluble dietary fiber at different ratios: Hydration characteristics, rheological properties, and ameliorative effects on constipation. Food Chemistry: X, 24, 101996. 39634524 10.1016/j.fochx.2024.101996 PMC11616548

[phy270467-bib-0080] Wang, Y. , Jiang, H. , Wang, L. , Gan, H. , Xiao, X. , Huang, L. , Li, W. , & Li, Z. (2023). Luteolin ameliorates loperamide‐induced functional constipation in mice. Brazilian Journal of Medical and Biological Research, 56, e12466. 36722660 10.1590/1414-431X2023e12466 PMC9883005

[phy270467-bib-0081] Yakubu, M. T. , & Adams, M. D. (2025). HPLC profile, biochemical and histoarchitectural changes in loperamide‐induced constipated Wistar rats after oral administration of aqueous extract of Cnestis ferruginea (Vahl ex DC) roots. Pharmacological Research‐Natural Products, 6, 100144.

[phy270467-bib-0082] Yang, S. , & Yu, M. (2021). Role of goblet cells in intestinal barrier and mucosal immunity. Journal of Inflammation Research, 14, 3171–3183. 34285541 10.2147/JIR.S318327 PMC8286120

[phy270467-bib-0083] Zhang, H. , & Tsao, R. (2016). Dietary polyphenols, oxidative stress and antioxidant and anti‐inflammatory effects. Current Opinion in Food Science, 8, 33–42.

